# Genome-wide association study revealed a promising region and candidate genes for eggshell quality in an F_2_ resource population

**DOI:** 10.1186/s12864-015-1795-7

**Published:** 2015-07-31

**Authors:** Congjiao Sun, Liang Qu, Guoqiang Yi, Jingwei Yuan, Zhongyi Duan, Manman Shen, Lujiang Qu, Guiyun Xu, Kehua Wang, Ning Yang

**Affiliations:** National Engineering Laboratory for Animal Breeding and MOA Key Laboratory of Animal Genetics and Breeding, College of Animal Science and Technology, China Agricultural University, Beijing, 100193 China; Jiangsu Institute of Poultry Science, Yangzhou, Jiangsu 225125 China

**Keywords:** GWAS, Eggshell quality, Age-dependent traits, SNP-based heritability, Chicken

## Abstract

**Background:**

Eggshell is subject to quality loss with aging process of laying hens, and damaged eggshells result in economic losses of eggs. However, the genetic architecture underlying the dynamic eggshell quality remains elusive. Here, we measured eggshell quality traits, including eggshell weight (ESW), eggshell thickness (EST) and eggshell strength (ESS) at 11 time points from onset of laying to 72 weeks of age and conducted comprehensive genome-wide association studies (GWAS) in 1534 F_2_ hens derived from reciprocal crosses between White Leghorn (WL) and Dongxiang chickens (DX).

**Results:**

ESWs at all ages exhibited moderate SNP-based heritability estimates (0.30 ~ 0.46), while the estimates for EST (0.21 ~ 0.31) and ESS (0.20 ~ 0.27) were relatively low. Eleven independent univariate genome-wide screens for each trait totally identified 1059, 1026 and 1356 significant associations with ESW, EST and ESS, respectively. Most significant loci were in a region spanning from 57.3 to 71.4 Mb of chromosome 1 (GGA1), which together account for 8.4 ~ 16.5 % of the phenotypic variance for ESW from 32 to 72 weeks of age, 4.1 ~ 6.9 % and 2.95 ~ 16.1 % for EST and ESS from 40 to 72 weeks of age. According to linkage disequilibrium (LD) and conditional analysis, the significant SNPs in this region were in extremely strong linkage disequilibrium status. Ultimately, two missense SNPs in GGA1 and one in GGA4 were considered as promising loci on three independent genes including *ITPR2, PIK3C2G,* and *NCAPG.* The homozygotes of advantageously effective alleles on *PIK3C2G* and *ITPR2* possessed the best eggshell quality and could partly counteract the negative effect of aging process. *NCAPG* had certain effect on eggshell quality for young hens.

**Conclusions:**

Identification of the promising region as well as potential candidate genes will greatly advance our understanding of the genetic basis underlying dynamic eggshell quality and has the practical significance in breeding program for the improvement of eggshell quality, especially at the later part of laying cycle.

**Electronic supplementary material:**

The online version of this article (doi:10.1186/s12864-015-1795-7) contains supplementary material, which is available to authorized users.

## Background

A vast number of eggs are produced annually for human consumption worldwide. Avian calcified eggshell as a unique bioceramic material can provide protection to egg contents from physical damage and microorganic invasion [1, 2]. Changes in eggshell properties are directly related to increasing risk of foodborne disease for the consumer [3]. Low eggshell quality will also lead to more cracked eggs in the automatic sorting and packing process in the modern egg industrial production [4]. It has been estimated that improving the mean eggshell strength by one Newton will lead to 0.5 % less broken eggs per hen per laying cycle [5]. On the other hand, eggshell is biologically significant for bird embryo developments by allowing gas exchange between the embryo and the environment and supplying calcium for the embryo bone deposition [6, 7]. Hatching eggs with thin eggshell have high embryonic mortality, owing to more loss of water vapour during the incubation [8]. Furthermore, eggshell are subject to quality loss with the aging process of laying hens [9], which hinders the developmental trend to prolong the laying cycles of egg-type chicken in the future. Therefore, understanding the genetic control for dynamic eggshell quality with aging process is of great economic and biological importance.

In recent years, the genomic [5, 10], transcriptomic [11–13], proteomic [14–18] and structural analyses [19–21] of eggshell have been carried out to provide new insights into better understanding on the eggshell mineralization and its ultrastructure or micostructure that contribute to eggshell quality. Identifying the quantitative trait loci (QTLs) that relate to eggshell quality is one of the most powerful strategies to illustrate the genetic mechanism underlying eggshell quality. Up to date, a total of 62 QTLs related to eggshell quality have been collected in the AnimalQTLdb (http://www.animalgenome.org/cgi-bin/QTLdb/index). However, most previously reported QTLs were identified by low-density linkage analysis with limited markers of microsatellites [22–24], which restricted the confidence intervals and mapping accuracy [25, 26]. Recently, genome-wide association study (GWAS) has been utilized to identify the associations between genomic loci and phenotypes with relatively high-density SNP arrays in chicken [27]. For instance, Liu et al. [10] conducted the first GWA analysis with relatively high-density SNA array (60 K) to refine the associations with egg production and egg quality traits in chicken. With the rapid advance in next-generation sequencing technologies, large amounts of SNPs in chicken have been discovered [28]. The development of 600 K Affymetrix Chicken SNP array allows more efficient screening of the causal loci and genes in relevance to target traits.

It is notable that many complex traits are dynamic varied with the aging process of animals [29, 30]. However, previous studies utilized the phenotypes from limited age points. Animal phenotypes at different age should be utilized in GWA analysis to refine the associations with age-dependent complex traits and increase the statistical power. Growing evidences had been provided that this type of “longitudinal design” could efficiently identify the time-dependent or consistent loci for complex traits [31, 32].

In the current study, we conducted GWA analysis on the dynamic eggshell quality traits at 11 time points from onset of laying to 72 weeks old utilizing a 600 K high-density SNP arrays in an F_2_ chicken population. We aimed to explore the associated genomic loci and genes that contribute to the phenotypic variability and dynamic change in eggshell quality traits, and to provide potential breeding tools for the improvement of eggshell quality.

## Results

### Phenotype description and genetic parameters

Means and standard deviations for eggshell quality traits, including ESW, EST and ESS, at 11 time points from onset of laying to 72 weeks of age are presented in Table 1 and Additional file 1: Figure S1. These three eggshell quality traits displayed a smooth curve with hen age except 36 weeks-old, at which ESW, EST and ESS showed a abrupt decrease (Additional file 1: Figure S1) and the results of GWA analysis in this week were also unusual (Table 3). We speculated that this might be caused by the feed or environment changes in this period leading to eggshell quality decrease. For this reason, phenotypes collected at 36 weeks-old were not included in the subsequent multivariate GWA analysis. The additive genetic variations of ESW, EST and ESS at different age were estimated from all eligible markers using GCTA. ESW at all ages exhibited moderate SNP-based heritability estimates (0.30 ~ 0.46, Table 2), and the highest SNP-based heritability estimate was found in ESW44 (*h*_*snp*_^2^ = 0.46). However, the estimates for EST (0.21 ~ 0.31, Additional file 2: Table S1) and ESS (0.20 ~ 0.27, Additional file 2: Table S2) were relatively low. Moreover, bivariate GCTA analyses indicated that ESW at different age were highly and positively correlated, specially at neighboring time points (*r*_*g*_ > 0.90).

**Table 1:** Descriptive statistics of eggshell traits in the *F*_*2*_ population

Age (wks)	N	Eggshell traits (Mean ± SD)		
		ESW (g)	EST (mm)	ESS (kg/cm^2^)
AFE	1494	3.69 ± 0.36	0.393 ± 0.017	3.61 ± 0.57
32	1473	4.40 ± 0.45	0.402 ± 0.021	3.70 ± 0.67
36	1464	4.37 ± 0.55	0.396 ± 0.022	3.59 ± 0.75
40	1476	4.64 ± 0.50	0.401 ± 0.021	3.67 ± 0.72
44	1420	4.63 ± 0.55	0.399 ± 0.023	3.60 ± 0.81
48	1226	4.70 ± 0.59	0.401 ± 0.024	3.51 ± 0.86
52	1225	4.68 ± 0.59	0.396 ± 0.024	3.43 ± 0.86
56	1348	4.74 ± 0.61	0.395 ± 0.024	3.40 ± 0.89
60	1364	4.70 ± 0.65	0.392 ± 0.024	3.25 ± 0.92
66	1304	4.62 ± 0.64	0.387 ± 0.028	3.17 ± 0.93
72	1253	4.57 ± 0.62	0.395 ± 0.025	3.02 ± 0.80

*N* number of samples, *ESW* eggshell weight, *EST* eggshell thickness, *ESS* eggshell strength, *Mean* arithmetic mean, *SD* standard deviation, *AFE* age of first egg

**Table 2:** Summary of genetic analysis for eggshell weights at different wks of age

Traits^a^	FESW	ESW32	ESW36	ESW40	ESW44	ESW48	ESW52	ESW56	ESW60	ESW66	ESW72
FESW	0.41(0.04)	0.87(0.04)	0.87(0.05)	0.77(0.06)	0.83(0.06)	0.77(0.06)	0.77(0.06)	0.66(0.07)	0.70(0.07)	0.68(0.07)	0.65(0.07)
ESW32	0.53	0.30(0.04)	1.00(0.03)	0.99(0.02)	0.96(0.03)	0.93(0.03)	0.96(0.03)	0.87(0.05)	0.90(0.04)	0.89(0.04)	0.87(0.05)
ESW36	0.41	0.57	0.41(0.04)	1.00(0.03)	0.98(0.04)	0.98(0.04)	0.99(0.04)	0.92(0.05)	1.00(0.04)	0.92(0.05)	0.93(0.05)
ESW40	0.46	0.61	0.55	0.37(0.04)	1.00(0.02)	0.99(0.02)	0.97(0.03)	0.92(0.04)	0.95(0.03)	0.96(0.03)	0.92(0.03)
ESW44	0.39	0.61	0.51	0.64	0.46(0.04)	1.00(0.02)	1.00(0.02)	0.98(0.03)	0.98(0.03)	0.96(0.03)	0.96(0.03)
ESW48	0.38	0.57	0.51	0.61	0.64	0.45(0.05)	1.00(0.02)	0.98(0.03)	1.00(0.03)	0.98(0.03)	0.95(0.03)
ESW52	0.40	0.58	0.52	0.62	0.65	0.69	0.39(0.05)	1.00(0.02)	0.99(0.03)	0.97(0.03)	0.95(0.03)
ESW56	0.35	0.52	0.45	0.58	0.61	0.62	0.65	0.33(0.05)	1.00(0.03)	0.95(0.03)	0.93(0.03)
ESW60	0.33	0.49	0.44	0.56	0.54	0.57	0.63	0.62	0.44(0.04)	0.98(0.03)	0.96(0.03)
ESW66	0.36	0.54	0.47	0.58	0.59	0.62	0.65	0.66	0.64	0.40(0.05)	0.99(0.02)
ESW72	0.38	0.56	0.47	0.61	0.63	0.61	0.68	0.67	0.63	0.74	0.43(0.05)

Diagonal: heritability estimates. Lower triangle: phenotypic correlations. Upper triangle: genetic correlations. Standard errors of the estimates are in parentheses

^a^ *FESW* eggshell weight of first egg, *ESW32, ESW36, ESW40, ESW44, ESW48, ESW52, ESW56, ESW60, ESW66, ESW72* eggshell weights of 32,36,40,44,48,52,56,60,66,72 weeks of age

### Identification of candidate loci by GWAS

**Fig. 1:**
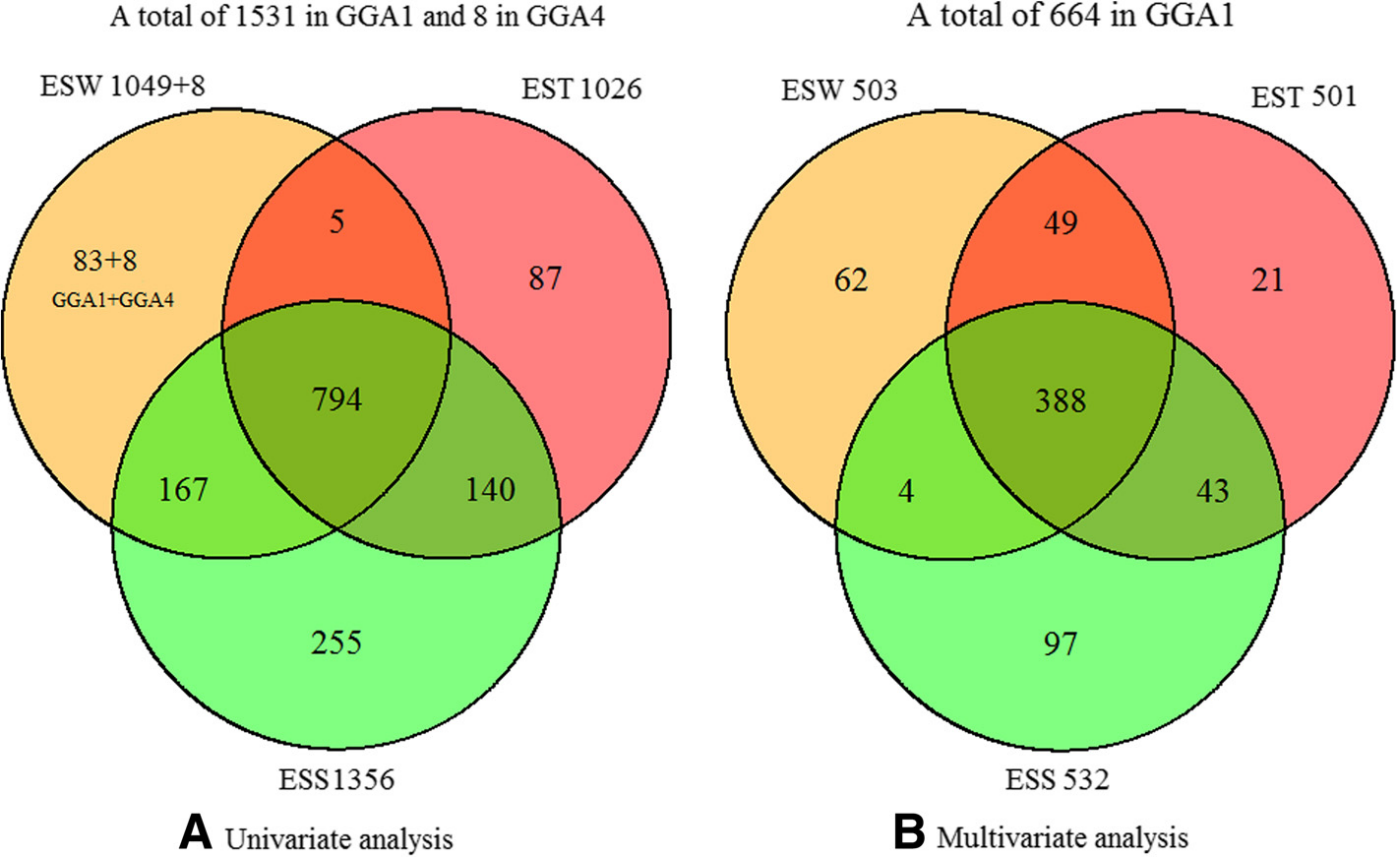
Venn diagram of significant SNPs associated with three eggshell quality traits by univariate (**a**) and multivariate (**b**) model. Eggshell weight, eggshell thickness and eggshell strength were abbreviated as ESW, EST and ESS respectively

**Fig. 2:**
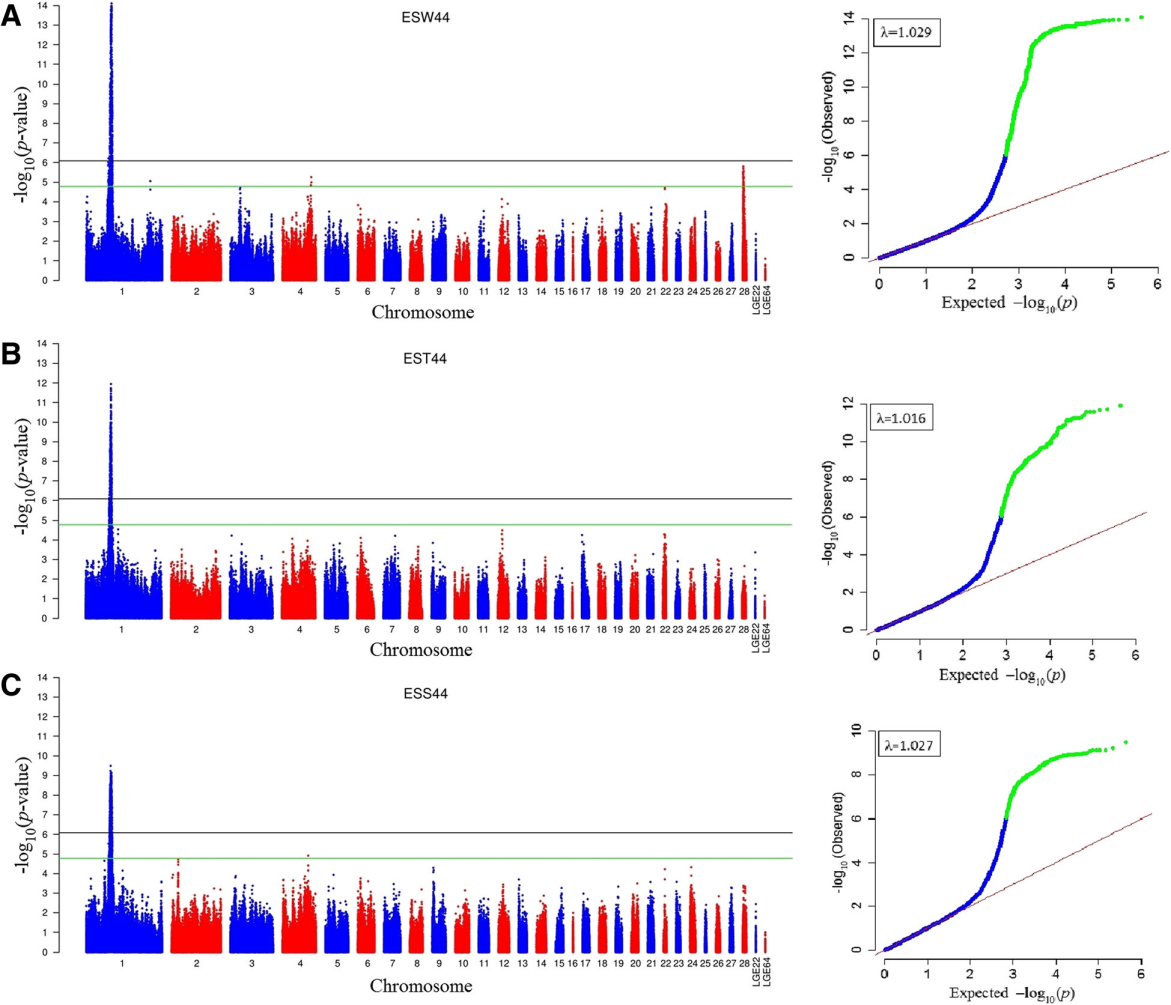
Manhattan plot (left) and quantile-quantile plot (right) of the observed *P*-values for ESW (**a**), EST (**b**) and ESS (**c**) at 44 weeks of age (ESW44). The Manhattan plot indicates -log_10_ (observed *P*-values) for genome-wide SNPs (y-axis) plotted against their respective positions on each chromosome (x-axis), and the horizontal green and black lines depict the genome-wide suggestive (1.69 × 10^−5^) and significant (8.43 × 10^−7^) threshold, respectively. For quantile-quantile plot, the x-axis shows the expected -log_10_-transformed *P*-values, and the y-axis represents the observed -log_10_-transformed *P*-values. The genomic inflation factors (λ) are shown on the top left in the QQ plot. Green points represent the genome-wide significant associations

Eggshell quality traits were collected at 11 time points from onset of laying to 72 weeks. At each time point, three separate genome-wide association tests were conducted for each eggshell quality trait with univariate method. A total of 1057, 1026 and 1356 genome-wide significant associations were identified with ESW, EST and ESS, respectively (Table 2, Additional file 2: Table S3). Almost all the significant locus were in a region spanning from 57.3 to 71.4 M of chromosome 1 (GGA1) (Table 3). Out of these loci in this region, 794 SNPs were observed in association with all these three eggshell quality traits at genome-wide significance level (Fig. 1). It was notable that most of the SNPs in this region possess a MAF more than 0.3. In addition, 8 loci on chromosome 4 (GGA4) were significantly associated with ESW, but only for young hens from age of first egg (AFE) to 40 week old. The global view of the putative *P-*values for all SNPs affecting eggshell quality traits at 44weeks is given in Fig. 2, and the Manhattan and quantile-quantile (QQ) plots for the remaining time points in Additional file 1: Figure S2.

**Table 3:** Number and distribution of significant SNPs for eggshell traits

Age	ESW		EST	ESS
	GGA1	GGA4	GGA1	GGA1
AFE	0	1	0	0
32	590	2	0	230
36	48	7	0	121
40	867	3	501	809
44	814	0	579	624
48	569	0	167	378
52	585	0	637	596
56	601	0	618	608
60	615	0	722	870
66	717	0	714	1016
72	633	0	721	1091
total (Region)	1049 (60.1 ~ 69.0 M)	8	1026 (59.7 ~ 70.4 M)	1356 (57.3 ~ 71.4 M)
Multi-analysis^a^ (Region)	503 (62.4 ~ 67.9 M)	0	501 (60.8 ~ 67.8 M)	532 (60.5 ~ 67.0 M)

*AFE* age of first egg, *ESW* eggshell weight, *EST* eggshell thickness, *ESS* eggshell thickness

^a^Multi-analysis means using multivariate model for GWA analysis. For the analysis of ESW and ESS, time points of 32 weeks, and 40–72 weeks were used; for EST, time points of 40–72 weeks were used

To enhance the statistical power, a joint GWA analysis of multi time points was performed by fitting these SNPs into a multivariate model. The time points that had significant SNPs were included in this model except for 36 weeks owing to the abnormal phenotype in this week. The 1531 significant SNPs related to eggshell quality identified by univariate method in GGA1 were further analyzed in this multivariate model. Consequently, a total of 503, 501 and 532 significant hits on GGA1 from 60.5 to 67.9 M presented consistent and compelling associations with longitudinal eggshell quality traits (Table 3, Additional file 2: Table S4), and 388 SNPs out of these locus had pervasive effect on ESW, EST and ESS (Fig. 1b).

### Linkage disequilibrium (LD) and conditional analysis

**Fig. 3:**
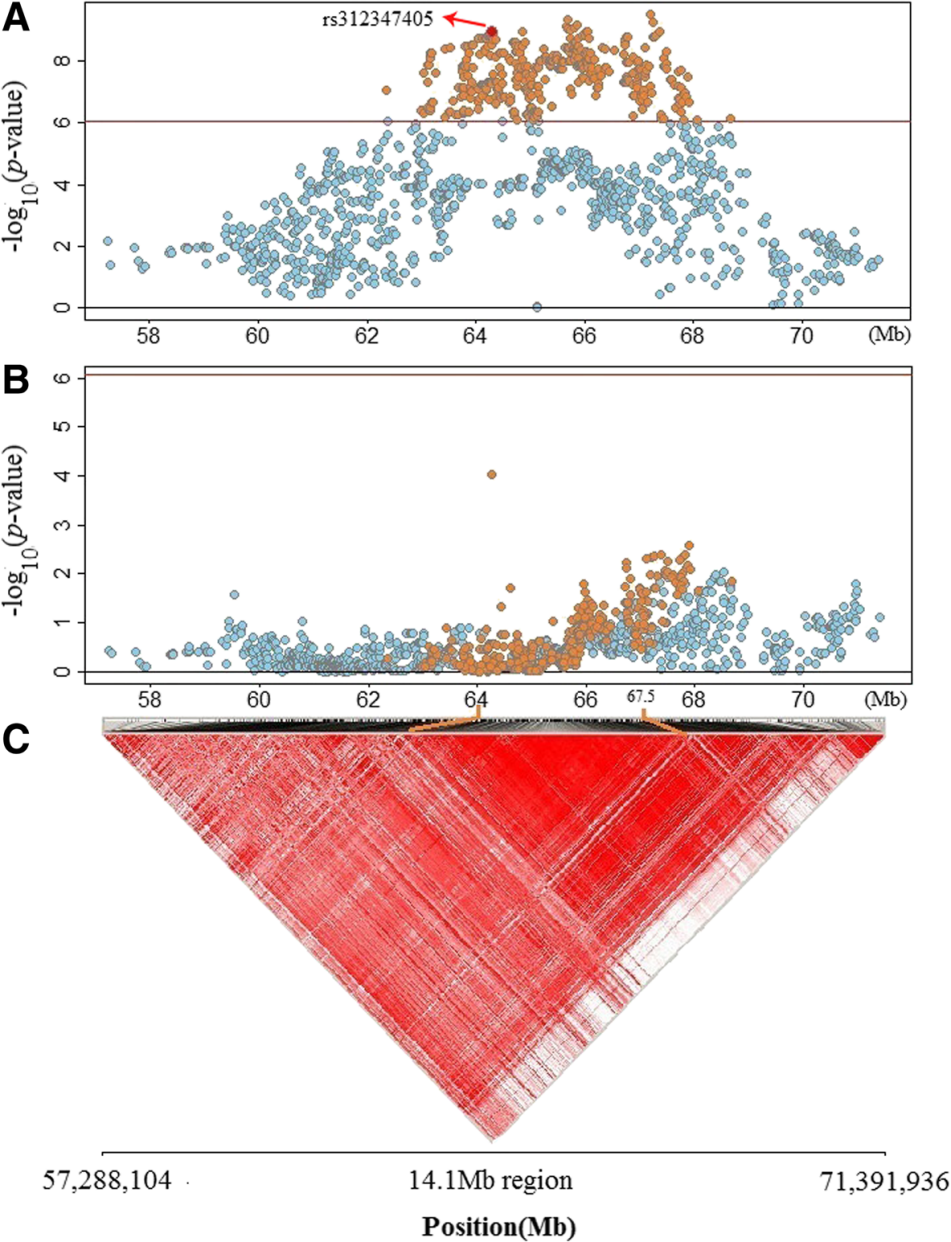
Regional plot and conditional analysis in multivariate model for ESW. Plot **a**: Up to 1531 significant SNPs (orange and blue points) obtained by univariate model were re-analyzed for their association with ESW by multivariate model (multiple time points). The -log_10_ (observed *P*-values) of SNPs (y-axis) are presented according to their chromosomal positions (x-axis). Totally 503 SNPs (orange points) reached genome-wide significance level (black line, 8.43 × 10^−7^). Plot **b**: The genotype of *rs312347405* was put into the multivariate model as covariances for conditional analysis. After conditioning on *rs312347405,* the significant SNPs in plot A (orange points) were all substantially attenuated below genome-wide significant level in plot B. Plot **c**: regional plot shows extremely strong linkage disequilibrium status exist in this 14.1 Mb region, especially for the LD block from 64.0 to 67.5 Mb

The linkage disequilibrium (LD) analysis were then carried out and the results showed the uncovered SNPs in GGA1 were in extremely strong linkage disequilibrium status, especially for the LD block from 64.0 to 67.5 Mb that include half (732/1531) of the total loci (Fig. 3). To detect the independent acting locus in this region, we then carried out stepwise conditional GWA analysis to prioritize separately associated SNPs. At locus *rs312347405,* a missense mutation in association with all three eggshell traits was then fitted into the multivariate model using its genotype as covariate to explore the change of the associations with other loci. As shown in Fig. 3, if the genotype of *rs312347405* was used as covariate in multivariate analysis, the significance levels of all other loci were substantially attenuated below genome-wide significant level. Regional plots and conditional analysis in multivariate model for the traits of eggshell thickness (EST) and eggshell strength (ESS) were shown in Additional file 1: Figure S3.

### Estimation of contribution to phenotypic variation (CPV)

The phenotypic variance explained by loci or genomic region were estimated by a tool of GCTA for three eggshell traits. All together, 1531 significant loci from 57.3 to 71.4 Mb in GGA1 could account for 8.4 ~ 16.5 % of the phenotypic variance for ESW from 32 to 72 weeks of age, 4.1 ~ 6.9 % and 2.95 ~ 16.1 % for EST and ESS from 40 to 72 weeks of age. For single locus, five missense mutations out of these 1531 loci were estimated for their CPVs (Table 4). Among these five loci, two SNPs presented high CPVs for eggshell traits. One loci, *rs312347405*, could independently explain 5.25–8.7 %, 4.22–7.41 % and 3.18–7.59 % of the phenotypic variance for ESW, EST and ESS from 32 to 72 weeks of age respectively. Notably, the effect alleles at *rs314058619* were associated with ESW at each time point except the first egg. The other one, *rs316607577*, could account for 4.78–7.77 %, 3.42–5.31 % and 2.43–11.52 % of the phenotypic variance respectively.

**Table 4:** Contributions of five missense mutations and genomic regions to eggshell quality traits at different wk of age

SNP		*rs312347405*	*rs14837998*	*rs316793137*	*rs14840004*	*rs316607577*	*Region 1*
Chromosome		GGA1	GGA1	GGA1	GGA1	GGA1	GGA1
Position (bp)		64,287,542	65,472,759	67,081,703	67,315,919	67,961,420	57.3–71.4 Mb
Gene symbol		PIK3C2G	RECQL	ABCC9	CASC1	ITPR2	-
EA/AA^a^		C/G	A/G	A/G	T/G	C/T	-
MAF		0.485	0.353	0.414	0.386	0.413	-
Amino acid change		Leu/ Val	Cys/ Arg	Asn/ Asp	Thr/ Pro	Ger/ Gly	-
ESW32	beta (SE)^b^	−0.253 (0.048)	0.276 (0.051)	0.269 (0.049)	−0.151 (0.051)	0.246(0.052)	-
	CPV (%)	5.25	3.36	3.12	0.82	4.90	8.55
ESW40	beta (SE)	−0.361 (0.050)	0.320 (0.050)	0.349 (0.049)	−0.264 (0.050)	0.347(0.051)	-
	CPV (%)	7.21	4.79	5.67	3.08	4.78	12.5
ESW44	beta (SE)	−0.285 (0.046)	0.280 (0.052)	0.302 (0.049)	−0.265 (0.051)	0.291(0.051)	-
	CPV (%)	8.06	3.37	3.97	2.92	6.08	11.17
ESW48	beta (SE)	−0.286 (0.051)	0.228 (0.056)	0.297 (0.053)	−0.195 (0.055)	0.285(0.056)	-
	CPV (%)	7.31	2.33	4.26	1.71	4.93	8.45
ESW52	beta (SE)	−0.321 (0.051)	0.220(0.056)	0.292 (0.053)	−0.179 (0.055)	0.298(0.055)	-
	CPV (%)	7.51	2.21	3.86	1.31	7.77	18.54
ESW56	beta (SE)	−0.333 (0.050)	0.251 (0.053)	0.297 (0.021)	−0.124 (0.053)	0.269(0.053)	-
	CPV (%)	6.8	2.81	4.13	0.52	7.44	15.25
ESW60	beta (SE)	−0.368 (0.054)	0.276 (0.052)	0.295 (0.049)	−0.215 (0.051)	0.291(0.052)	-
	CPV (%)	6.64	3.53	4.11	2.01	5.74	13.77
ESW66	beta (SE)	−0.388 (0.046)	0.252 (0.055)	0.316 (0.052)	−0.234 (0.053)	0.316(0.054)	-
	CPV (%)	8.73	2.86	4.82	2.37	5.86	10.15
ESW72	beta (SE)	−0.382 (0.047)	0.252 (0.056)	−0.332 (0.053)	−0.211 (0.055)	0.292(0.056)	-
	CPV (%)	8.12	2.92	5.19	1.94	6.90	15.14
EST40	beta (SE)	−0.361(0.054)	0.245(0.052)	0.236(0.05)	−0.158(0.051)	0.198(0.053)	-
	CPV (%)	5.33	2.78	2.67	1.07	3.98	5.56
EST44	beta (SE)	−0.313(0.049)	0.211(0.051)	0.225(0.048)	−0.236(0.049)	0.222(0.049)	-
	CPV (%)	4.87	2.01	2.27	2.48	3.42	6.15
EST48	beta (SE)	−0.291(0.053)	0.19(0.054)	0.2(0.052)	−0.141(0.053)	0.199(0.053)	-
	CPV (%)	4.22	1.62	1.86	0.86	4.55	5.44
EST52	beta (SE)	−0.308(0.051)	0.2(0.054)	0.196(0.05)	−0.141(0.052)	0.168(0.052)	-
	CPV (%)	4.63	1.75	1.77	0.81	4.18	5.51
EST56	beta (SE)	−0.312(0.047)	0.222(0.05)	0.234(0.048)	−0.19(0.049)	0.181(0.05)	-
	CPV (%)	4.81	2.21	2.68	1.65	5.33	5.61
EST60	beta (SE)	−0.387(0.051)	0.245(0.053)	0.267(0.05)	−0.25(0.051)	0.212(0.052)	-
	CPV (%)	7.01	2.72	3.42	2.84	4.21	6.92
EST66	beta (SE)	−0.269(0.047)	0.203(0.052)	0.201(0.048)	−0.178(0.049)	0.171(0.05)	-
	CPV (%)	3.49	1.74	1.87	1.33	4.63	4.12
EST72	beta (SE)	−0.339(0.05)	0.165(0.054)	0.237(0.05)	−0.25(0.051)	0.217(0.052)	-
	CPV (%)	5.86	1.21	2.77	3.04	4.02	5.35
ESS32	beta (SE)	−0.253(0.048)	0.172(0.049)	0.129(0.047)	−0.128(0.047)	0.126(0.048)	-
	CPV (%)	3.18	1.28	0.74	0.73	2.65	2.95
ESS40	beta (SE)	−0.361(0.05)	0.245(0.05)	0.264(0.048)	−0.193(0.049)	0.267(0.05)	-
	CPV (%)	6.56	2.80	3.55	1.73	4.82	6.59
ESS44	beta (SE)	−0.285(0.046)	0.189(0.049)	0.185(0.047)	−0.165(0.048)	0.187(0.048)	-
	CPV (%)	4.04	1.57	1.63	1.21	2.43	4.11
ESS48	beta (SE)	−0.286(0.051)	0.152(0.054)	0.149(0.051)	−0.129(0.052)	0.183(0.052)	-
	CPV (%)	4.16	0.96	1.04	0.78	3.56	4.22
ESS52	beta (SE)	−0.321(0.051)	0.169(0.054)	0.241(0.05)	−0.17(0.052)	0.224(0.052)	-
	CPV (%)	5.30	1.20	2.97	1.39	5.62	5.26
ESS56	beta (SE)	−0.333(0.05)	0.217(0.052)	0.207(0.05)	−0.142(0.051)	0.21(0.052)	-
	CPV (%)	5.64	2.18	2.13	0.92	6.10	5.56
ESS60	beta (SE)	−0.368(0.054)	0.303(0.06)	0.318(0.055)	−0.233(0.058)	0.227(0.058)	-
	CPV (%)	6.70	4.09	4.68	2.29	11.52	16.12
ESS66	beta (SE)	−0.388(0.046)	0.226(0.052)	0.25(0.048)	−0.229(0.049)	0.304(0.049)	-
	CPV (%)	7.59	2.31	3.15	2.43	5.86	6.06
ESS72	beta (SE)	−0.382(0.047)	0.26(0.053)	0.271(0.049)	−0.239(0.051)	0.235(0.051)	-
	CPV (%)	7.35	3.06	3.62	2.70	5.16	6.75

*EA* effect allele (minor allele), *AA* alternative allele (major allele), *MAF* minor allele frequency, *CPV* contribution to phenotypic variance (%), *ESW32, ESW40, ESW44, ESW48, ESW52, ESW56, ESW60* eggshell weights of 32, 40, 44, 48, 52, 56, 60, 66, 72 weeks of age

^a^Estimated allelic substitution effect per copy of the effect allele (EA); *SE* standard error of the beta ^b^Beta means the effect size of minor alleles. Positive/negative effect size value means that the substitution of major allele to minor allele can lead to heavier/lighter yolk or ovary weight

**Fig. 4:**
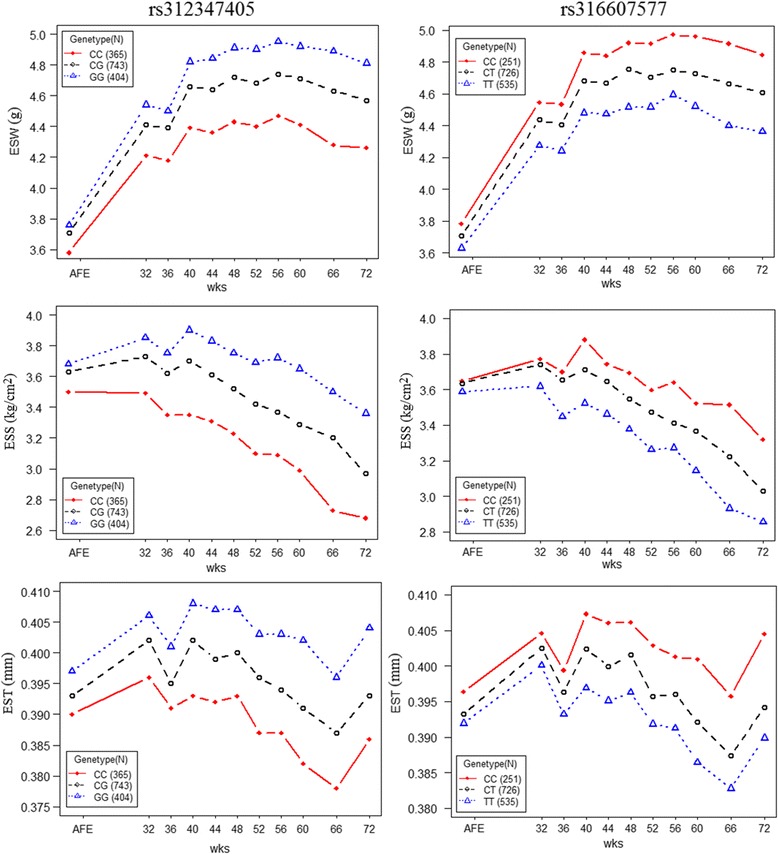
Phenotypic differences contributed by loci of *rs312347405* and *rs316607577* on genes of *PIK3C2G* and *ITPR2*. The left three plots describe the phenotypes of ESW, EST and ESS among three genotypes at *rs312347405*. The right three plots describe the phenotypes of ESW, EST and ESS among three genotypes at *rs316607577*. Red square, black circle and blue triangle denote minor-allele homozygotes, heteorozygotes and major-allele homozygotes, respectively. Number of samples for each genotype is indicated in the top or bottom left

The phenotypic differences among 3 genotypes at these two loci are displayed in Fig. 4. There was no difference in eggshell quality traits among different genotypes for the first egg. However, along with the aging process, profound differences in eggshell quality appeared among three genotypes. Eggshell of each genotype exerted different rate of quality decay with aging process, i.e., the homozygotes of advantageously effective alleles possessed the best eggshell quality and could partly counteract the negative effect of aging process. However, the change trends of eggshell traits among three genotypes were concordant along with age.

### Promising genes related to eggshell quality

Utilizing gene annotation of the causal locus allowed us to screen the putative genes relating to eggshell quality. For SNPs-traits association analysis, the missense mutations on exons were more meaningful than the SNPs located on introns or intergenic regions. We totally found five missense loci at GGA1 and one at GGA4 using univariate genome-wide screens. They located on 6 independent genes including phosphatidylinositol–4- phosphate 3-kinase, catalytic subunit type 2 gamma (*PIK3C2G*)*,* inositol 1,4,5-trisphosphate receptor type 2 (*ITPR2*)*,* RecQ helicase-like (*RECQL*)*,* ATP-binding cassette sub-family C member 9 (*ABCC9*) and cancer susceptibility candidate 1 (*CASC1*) on GGA1 and non-SMC condensin I complex subunit G (*NCAPG*) on GGA4. Nonetheless, only two SNPs, *rs312347405* and *rs316607577,* located on *PIK3C2G* and *ITPR2*, remained significantly associated with eggshell quality traits after multivariate GWA analysis. Furthermore, as illustrated above, these two SNPs could explain more phenotypic variance than others. Consequently, we first considered *PIK3C2G* and *ITPR2* as the primary candidate genes associated with eggshell quality. The missense mutation in GGA4 that located on *NCAPG* was posterior putative locus for its roles in affecting early eggshell weight. In addition to missense mutations on exons, the mutations on 3′ or 5′ UTRs were also of biological significance for genetic variation underlying complex traits. We aggregately discovered 15 mutations that located on 3′ or 5′ UTRs corresponding to 13 independent genes (Additional file 2: Table S5). According to functional annotation and database research, however, most of these genes had no direct relevance to eggshell calcification or calcium metabolism, expect for *rs316447591* which located on the 3′UTR of *ITPR2* gene. The detailed message of promising loci and the corresponding candidate genes are listed in Table 5.

**Table 5:** Putative genes associated with eggshell quality

Gene symbol^a^	Chr	Tag SNP	Position	Location	AA subtitution	SIFT^b^
PIK3C2G	GGA1	rs312347405	64,287,542	Exon 27 of 32	Leu1253Val	0.14
ITPR2	GGA1	rs316607577	67,961,420	Exon 25 of 56	Ser1072Gly	0.40
		rs316447591	67,808,349	3′UTR region	-	-
NCAPG	GGA4	rs14491030	75,486,534	Exon 14 of 21	Val674Ala	0.74

*Chr* chromosome, *AA* amino acid, *SIFT* Sorting intolerant from tolerant

^a^Identification of the gene according to Ensembl genes database 76

^b^SIFT is a program that predicts whether an amino acid substitution affects protein function. Small values means deleterious amino acid change

## Discussion

It is known that eggshells are subject to quality loss along with the age process [9]. In egg-type chicken production, there exists a trend to prolong the laying cycles [33], whereas the decline of eggshell quality for old hens proposes a substantial challenge for this development pattern. In the present study, we elucidated genomic architecture underlying the age-dependent dynamic eggshell quality, which has biological and practical significance.

The QTL number detected for eggshell quality was limited in previous studies. The low to medium heritability of the eggshell trait revealed by our results and other study [34] suggested the necessary use of large population for causal mutant identification. Our F_2_ population consisting of 1512 hens is the largest population used for eggshell quality GWA analysis so far, and therefore the novel genomic region and loci revealed by the current study should be accurate and reliable. Our GWA analysis for dynamic eggshell quality traits identified thousands of significant associations, that were much more than those screened by previous GWA studies in chicken [10, 35]. These significant mutations were almost in a same region with an extremely strong linkage disequilibrium status, which may result from the insufficient recombination events in the F_2_ segregation population [36, 37]. Lots of QTL regions affecting ESW, EST and ESS were discovered by previous linkage studies [38] and mainly distributed on GGA 1–9 and GGA11. The promising genomic region from 57.3 to 71.4 Mb identified in the current study had no overlap with the previously reported regions in GGA 1, mainly on 105.2–107.3 Mb, 172.4–173.5 Mb, 180.4–180.5 Mb and 191.5–191.6 Mb (AnimalQTLdb).

The 1531 significant SNPs in this region were distributed on 116 independent genes. We tried to perform Gene Ontology (GO) [39] and pathway analysis [40] on these 116 genes, whereas no significant GO terms or pathways were enriched. This suggested there were not a series of functionally similar genes in this region to influence eggshell quality together. Most SNPs might passively display significant associations for their linkages to a sort of real causal mutants on one or a few genes. Hence, the phenotypic variance explained by all loci in this region was only slightly higher than single locus. It was notable that more associations were discovered for old hens (40–72 weeks) than young hens (AFE to 36 weeks), suggesting certain genetic variants were age-dependent. This was partially consistent with the other GWA studies, indicating no significant associations with both early and late shell quality [10, 41]. However, our results also proved the existence of common genetic variants consistently influencing eggshell quality from 40 to 72 weeks.

Six significant missense mutations in this genomic region were considered as the most important putative variants. After multivariate GWA analysis, only two loci, *rs312347405* and *rs316607577* on *PIK3C2G* and *ITPR2* in GGA1 remained significantly associated with dynamic eggshell quality traits. The loci of *rs312347405* on *PIK3C2G* could explain the most phenotypic variance of eggshell quality traits among the five missense mutations. Hens with homozygotes of advantageously effective alleles (GG) could produce eggs with excellent ESS, which decreased relatively within a narrow range along with the aging process. *PIK3C2G* encoded proteins belonging to the phosphoinositide 3-kinase (PI3K) family, containing a lipid kinase catalytic domain as well as a C-terminal C2 domain [42], which acted as calcium-dependent phospholipid binding motifs [43]. Previous proteomic screens revealed that a high proportion of lipid-binding proteins abundantly existed in eggshell matrix such as extracellular fatty acid-binding protein (Ex-FABP), prosaposin and apolipoprotein D [14]. Considering the low abundance of lipids in eggshell, the existence of high abundance of lipid-binding proteins in eggshell matrix was perplexed [14]. Furthermore, one association analysis revealed another lipid-related gene, low-density lipoprotein receptor-related protein 8 (LRP8) gene, as a new member of eggshell matrix protein, was significantly associated with eggshell traits [44]. Now in the current study *PIK3C2G* possessing the C2 domain acting as lipid binding motif was also implicated in eggshell property. The role of lipid binding proteins in eggshell or during eggshell formation should be re-recognized. *PIK3C2G* possessing the C2 domain could mediate translocation of proteins to lipid membranes, and also regulate protein-protein interactions in human and mammals [45]. The interoperable matrix proteins and calcite together formed the bioceramic eggshell [46]. It could be hypothesized that lipid binding proteins might act as carrier or modifier of matrix precursors, which mediate eggshell calcification process or act as frame proteins deposited into eggshell structure. Consequently, *PIK3C2G* was considered as candidate gene for eggshell quality.

The gene of inositol 1,4,5-trisphosphate receptor type 2 (*ITPR2*) harboring the loci of *rs316607577* (exon 25) was the positional and functional candidate gene for eggshell quality revealed by the current GWA analysis. The mutation of *rs316607577* was a nonconservative substitution of serine by glycine (S1072G), with the glycine-encoding allele being associated with stronger eggshell. A gene could be inactivated by a mutation either in a control site or in a coding region [47]. So *rs316447591* on 3′URT of *ITPR2* was also the susceptibility locus. *ITPR2* was known for its mediation in endoplasmic reticulum (ER) calcium release and inositol 1,4,5-trisphosphate (IP3) could mobilize Ca^2+^ from intracellular calcium stores to many types of cells [48]. The existence of *ITPR2* in uterine epithelial cell of chicken had been verified [11, 12], furthermore, the expression of *ITPR2* in uterus during eggshell calcification was significantly higher than that in magnum and duodenum which also possess active calcium metabolism [49]. This provided the evidences that *ITPR2* play roles in the regulation of intra-cellular Ca^2+^ transportation in uterus and contributed to the process of eggshell calcification. Recently, a genome-wide association study in humans identified *ITPR2* as a susceptibility gene for Kashin-Beck disease which is a chronic osteochondropathy [50], mainly characterized by cartilage degeneration, cartilage matrix degradation, chondrocyte necrosis and apoptosis [51]. This uncovered association with bone disease indicated that it might play a role in general mineralization processes. Birds process medullary bone, a nonstructural type of woven bone, to act as a reservoir for the minerals required for shell calcification [52]. Our previous comparative proteomic analysis for uterine fluid and eggshell matrix proteins also identified that many bone-development related proteins were up-presented in strong eggshell group [15]. Up to date, there only existed one paper referred to the association of ion transporter genes with eggshell quality. They found one SNP on a sodium channel gene (*SCNN1b*) had effects on eggshell strength [53], however, the phenotypic variance of ESS it could explained was relatively low (1.14 %) compared to that of *ITPR2* (2.43–11.52 %) in the current study. All the above evidences suggested *ITPR2* should be a crucial and promising candidate gene relating to eggshell calcification as well as eggshell mechanical property.

Another missense mutation (*rs14491030*) located on non-SMC condensin I complex subunit G (*NCAPG*) gene in GGA4 was discovered in association with eggshell weight for young hens in our GWA analysis. Many other studies reported multiple genomic regions containing this gene were identified to be associated with body weight [23, 54] and egg weight [35, 55] in chickens and many other growth traits in livestocks [56, 57]. The current study suggested that *NCAPG* also involved in influencing eggshell weight. Larger eggs generally owned heavier eggshell. Hence we could not exclude the potential cause that the significant association of *NCAPG* with ESW was just due to its relevance to egg weight. Nevertheless, we still considered *NCAPG* as a candidate gene for its consistent association with eggshell weight from 32 to 40 week.

Previous studies hypothesized that eggshell organic matrix as a complicated mixture of proteins might play a regulatory role in assembly of the calcite zone with calcium carbonate to ultimately determine its strength, and many evidences had been proposed to support this [17], such as the different matrix protein concentrations in strong and weak eggshells [15, 58]. Furthermore, many works conducted to investigate the associations between polymorphisms in genes encoding eggshell matrix proteins and eggshell quality traits [59, 60]. Takahashi *et al*. [60] conducted association analysis between ovocalyxin-32 gene haplotypes and eggshell quality traits in an F_2_ intercross and Dunn *et al.* [59] carried out association analysis between polymorphisms in 10 eggshell organic matrix genes and eggshell quality measurements in two lines. These two studies found some significant associations, however, the loci could only explain limited phenotypic variances. Our current study discovered no association between SNPs on eggshell matrix genes and eggshell quality, suggesting that the influences of eggshell matrix genes on eggshell quality were not caused by their nucleotide polymorphisms.

## Conclusions

The dynamic eggshell quality traits at 11 time points from onset of laying to 72 weeks were collected and used for genome-wide association analysis with a 600 K high-density SNP array. According to univariate and multivariate GWA analysis, we discovered a genomic region spanning from 57.3 to 71.4 M in GGA1 significantly associated with eggshell quality. LD and conditional analysis suggested this region were in extremely strong linkage disequilibrium status, especially for the LD block from 64.0 to 67.5 Mb that almost include half (732/1531) of the total significant loci. Ultimately, three genes, *PIK3C2G, ITPR2* and *NCAPG* identified from three missense mutations were considered as promising candidate genes that implicated in dynamic eggshell quality. The homozygotes of advantageously effective alleles on *PIK3C2G* and *ITPR2* possessed the best eggshell quality and could partly counteract the negative effect of aging process. These promising loci and genes could be helpful to engineer practical breeding programs for the improvement of eggshell quality for old hens to meet the need of prolonging the laying cycle. The new findings also have greatly advanced our understanding of the genetic basis underlying eggshell quality.

## Methods

### Resource population

The reciprocal crosses between a standard breed White Leghorn (WL) and a Chinese indigenous strain Dongxiang chickens (DX) were utilized to develop an F_2_ resource population. For parents, six WL and six DX males were initially mated with 133 DX and 80 WL females and generated 1029 and 552 chicks for F_1_ generation, respectively. Then 25 males and 407 females from WL/DX cross and 24 cocks and 235 hens from DX/WL cross in F_1_ generation were used to produce the F_2_ population, consisting of 3749 chicks in a single hatch originating from 49 half-sib and 590 full-sib families. The hens were kept in individual cages of the same environment with food and water *ad libitum* at the Jiangsu Institute of Poultry Sciences. Finally, 1534 hens from 49 half-sib families and 550 full-sib families with sufficient phenotypic and pedigree information were selected for SNP genotyping.

### Phenotypic measurements

Eggshell quality traits including eggshell weight (ESW), eggshell thickness (EST) and eggshell strength (ESS) were measured for the first egg of hens and then at 32, 36, 40, 44, 48, 52, 56, 60, 66 and 72 weeks of age, consisting of 11 time points. Except for the first egg, fresh eggs were collected for 4 successive days to ensure two eggs per hen. Then breaking strength (pole to pole) of each egg was measured vertically with the Eggshell Force Gauge (Model-II, Robotmation, Tokyo, Japan). After that, eggs were broken to remove the internal contents and the remaining eggshells were washed clean with tap water. After drying in the air at room temperature, eggshell weights and eggshell thickness were measured with electronic scale and eggshell thickness gauge (FHK, Tokyo, Japan) respectively. Descriptive statistics were calculated with R project (R version 3.1.2) using all available records. The function of ‘rntransform’ in the GenABEL package of R project was used for the rank-based inverse normal transformations (INTs) [61].

### Genotyping and quality control (QC)

Genomic DNA was extracted by standard phenol/chloroform method and genotyped with the 600 K Affymetrix Axiom Chicken Genotyping Array (Affymetrix, Inc. Santa Clara, CA, USA). Affymetrix Power Tools v1.16.0 (APT) software was then used for quality control and genotype calling. Specifically, only samples with dish quality control (DQC) > 0.82 and call rate > 97 % were included into the subsequent analyses. An R script supplied by Affymetrix was run to compute the SNP QC metrics and filter out individual SNPs falling below given thresholds. After these QC steps, 1512 individuals and 532,299 SNPs remained valid. Given the current statistical methods were more powerful for the detection of the associations between phenotypes and autosomal genotypes, all SNPs on sex chromosomes were excluded in QC process. The PLINK v1.90 package [62] were then used for further quality control to improve the detect power, in which SNPs with minor allele frequency (MAF) < 5 % and Hardy-Weinberg equilibrium (HWE) test *P <* 1 × 10^−6^ were removed from downstream analysis. Some sporadic missing genotypes were imputed using the BEAGLE v4.0 procedure [63], only SNPs with imputation quality score *R*^2^ > 0.5 were retained. Ultimately, up to 1512 individuals and 435,867 SNPs were valid for the following GWA analysis.

### Genome-wide Association (GWA) analysis

Principal component analysis (PCA) implemented in PLINK package was first conducted prior to GWA analysis to eliminate spurious associations resulting from the presence of cryptic relatedness or hidden population stratification. To properly decide the thresholds for genome-wide significant/suggestive associations, a method of simpleM [64] was used to corrected the number of multiple tests. With this approach, an effective number of 59,308 independent tests was suggested, hence the genome-wide significant and suggestive *P*-values were 8.43 × 10^−7^ (0.05/59,308) and 1.69 × 10^−5^ (1.00/59,308), respectively.

Univariate association test equipped with an exact mixed model approach in GEMMA v0.94 software [65] was first performed with the valid individuals and SNPs. The centered relatedness matrix was calculated by those independent SNPs, and then the derived Wald test *P*-value was calculated for the significance level between SNPs and phenotypes. The univariate linear mixed model is as follows:

$$ \mathbf{y}=\mathbf{W}\boldsymbol{\upalpha } +\mathbf{x}\beta +\mathbf{u}+\boldsymbol{\upvarepsilon} $$

**y** is an *n* × 1 vector of phenotypic values for *n* individuals, **W** is an *n* × *c* matrix of covariates (fixed effects, *i.e*., top five PCs) including a column vector of 1, **α** is a *c* × 1 vector of corresponding coefficients including the intercept, **x** is an *n* × 1 vector of marker genotypes at the locus tested, *β* is the corresponding effect size of the marker and all effects are reported for the minor allele in each marker, **u** is an *n* × 1 vector of random polygenic effects with a covariance structure as **u** ~ N (0,**K**V*g*), where **K** represents a known *n* × *n* genetic relatedness matrix derived from SNP markers and V*g* is the polygenic additive variance, and ε is an *n* × 1 vector of random residuals with ε ~ N (0,**I**V_*e*_), where **I** is an *n* × *n* identity matrix, and V_e_ is the residual variance component. We used the Wald test statistic ${\mathrm{F}}_{\mathrm{Wald}}={\widehat{\beta}}^2/\mathrm{V}\mathrm{a}\mathrm{r}\left(\widehat{\beta}\right)$ for each SNP to test the null hypothesis *β* = 0, where the best linear unbiased estimate (BLUE) of *β* and the corresponding sampling variance $Var\left(\widehat{\beta}\right)$ are obtained by solving the mixed model equations (MME) based on estimated V*g* and V_e_.

The manhattan plots and quantile-quantile (QQ) plots were drawn by the “gap” packages [66] in R project. The genomic inflation factor λ which were the judgements for the extent of false positive signals was also calculated with the function of estlambda implemented in the GenABEL package [61] in R project.

To further analyze genetic variants affecting the dynamic eggshell quality traits along with aging process, a multivariate association analysis [67] that directly utilized phenotypes from multiple time points on an individual was applied. Only the time points and SNPs that had significant associations in univariate model were also included in this multivariate model. For each SNP marker, a multivariate linear mixed model could be fitted in the following form:

$$ \mathbf{Y}=\mathbf{W}\mathbf{A}+\mathbf{x}{\beta}^T+\mathbf{G}+\mathbf{E} $$

where **Y** is an *n* by *d* matrix of *d* phenotypes for *n* individuals, **W** = (**w**_1_, ⋅ ⋅⋅, **w**_**c**_) is an *n* × *c* matrix of covariates (fixed effects, *i.e*., top five PCs) including a column of 1 s, **A** is a *c* by *d* matrix of corresponding coefficients including the intercept, **x** is an *n*-vector of marker genotypes, *β* is a *d* vector of marker effect sizes for the *d* phenotypes. It should be noted that **G** is an *n* by *d* matrix of random effects with **G** ∼ MVN_*n* × *d*_(0, **K**, **V**_*g*_) ∼ MVN_*n* × *d*_(0, **K**, **V**_*g*_) where **V**_*g*_ is a *d* by *d* symmetric positive definite matrix of genetic variance component, and **E** is an *n* by *d* matrix of residual errors with E ∼ MVN_*n* × *d*_(0, **I**, **V**_*e*_) where **V**_*e*_ is a *d* by *d* symmetric positive definite matrix of residual variance component (**K** and **I** are the same in the two models). MVN_*n* × *d*_(0, **V**_1_, **V**_2_) denotes the *n* × *d* matrix normal distribution with mean 0, row covariance matrix **V**_1_ (*n* by *n*) and column covariance matrix **V**_2_ (*d* by *d*).

### Conditional and Linkage disequilibrium (LD) analysis

Notably, many SNPs maybe passively significant associated with target traits, resulting from their strong linkage to a really causal mutants. To demarcate independent association signals in a putative region, the conditional analyses as well as the LD analysis were both performed in multivariate and univariate models, through fitting the genotypes of one candidate signal as covariates [62]. In general, GWAS does not distinguish a genuine causal locus from those statistically significant loci within a strong LD region. Therefore, in order to characterize potential candidate genes responsible for a trait, we firstly conducted a LD analysis and inferred the haplotype blocks containing peak SNPs by Haploview v4.2 [68]. A block is derived using the solid spine algorithm, and defined as that the first and last SNPs in a region are in strong LD (D ' ≥ 0.8) with all intermediate SNPs.

### Estimation of heritability and contribution to phenotypic variance (CPV)

Univariate restricted maximum likelihood (REML) implemented in GCTA v1.24 program [69] was performed to estimate the heritability explained by the eligible SNPs (*h*_*snp*_^2^). We also quantified the pair-wise genetic and phenotypic correlations for each trait at multiple time points with the bivariate mixed model [70]. A genetic relationship matrix (GRM) derived from all genotyped SNPs on autosomes and two linkage groups was created, and the top five PCs calculated by the GCTA tool were included as covariates to account for the potential population structure. We then estimated the phenotypic variance contributed by these associated loci or genomic region.

### Gene identification

Variant Effect Predictor (VEP) and Biomart tools based on the Galgal4 assembly supported by Ensembl were used for the identification of candidate genes that the significant loci located on [71] and detection the genes within in a given genomic region [72].

## Additional files

Additional file 1:
**Figure S1.** Change curve of eggshell weight (ESW), eggshell percentage (ESP), eggshell thickness (EST) and eggshell strength (ESS) along with the age of laying hens. Plots A, B, C, D displayed the change curve of ESW, ESP, EST and ESS respectively. **Figure S2.** Manhattan plot (left) and quantile-quantile plot (right) of the observed *P*-values for ESW, EST and ESS at age of first egg and at 32, 36, 40, 48, 52, 56, 60, 66, 72 weeks of old. The Manhattan plot indicates -log_10_ (observed *P*-values) for genome-wide SNPs (y-axis) plotted against their respective positions on each chromosome (x-axis), and the horizontal green and black lines depict the genome-wide suggestive (1.69 × 10^−5^) and significant (8.43 × 10^−7^) threshold, respectively. For quantile-quantile plot, the x-axis shows the expected -log_10_-transformed *P*-values, and the y-axis represents the observed -log_10_-transformed *P*-values. The genomic inflation factors (λ) are shown on the top left in the QQ plot. Green points represent the genome-wide significant associations. **Figure S3.** Regional plots and conditional analysis in multivariate model for eggshell thickness (EST) and eggshell strength (ESS). Plot A: regional and conditional plot for EST. Plot B: regional and conditional plot for ESS. (PDF 4277 kb) Additional file 2:
**Table S1. Summary of genetic analysis for eggshell thickness at different wks of age. Table S2. Summary of genetic analysis for eggshell strength at different wks of age. Table S3.** Genome-wide significant SNPs for eggshell weight (ESW), eggshell thickness (EST) and eggshell strength (ESS) at different age by univariate model. **Table S4.** Genome-wide significant SNPs for eggshell weight (ESW),eggshell thickness (EST) and eggshell strength (ESS) by multivariate model. (XLSX 1372 kb). **Table S5.** Significant loci that located on 3’ or 5’ UTRs.

## Abbreviations

GWAS: Genome-wide association study
GWA: Genome-wide association
SNP: Single nucleotide polymorphism
QTL: Quantitative trait loci
LD: Linkage disequilibrium
REML: Restricted maximum likelihood
GRM: Genetic relationship matrix
MAF: Minor allele frequency
HWE: Hardy-Weinberg equilibrium
PCA: Principal component analysis
VEP: Variant effect predictor
