# Supplementary material for: Genome-wide association study revealed a promising region and candidate genes for eggshell quality in an F2 resource population
Source: BMC Genomics. 2015 Jul 31;16(1):565. doi: 10.1186/s12864-015-1795-7 (PMC4521446; doi:10.1186/s12864-015-1795-7)
Supplement: Additional file 1: — Figure S1. Change curve of eggshell weight (ESW), eggshell percentage (ESP), eggshell thickness (EST) and eggshell strength (ESS) along with the age of laying hens. Plots A, B, C, D displayed the change curve of ESW, ESP, EST and ESS respectively. Figure S2. Manhattan plot (left) and quantile-quantile plot (right) of the observed P-values for ESW, EST and ESS at age of first egg and at 32, 36, 40, 48, 52, 56, 60, 66, 72 weeks of old. The Manhattan plot indicates -log10 (observed P-values) for genome-wide SNPs (y-axis) plotted against their respective positions on each chromosome (x-axis), and the horizontal green and black lines depict the genome-wide suggestive (1.69 × 10^−5^) and significant (8.43 × 10^−7^) threshold, respectively. For quantile-quantile plot, the x-axis shows the expected -log10-transformed P-values, and the y-axis represents the observed -log10-transformed P-values. The genomic inflation factors (λ) are shown on the top left in the QQ plot. Green points represent the genome-wide significant associations. Figure S3. Regional plots and conditional analysis in multivariate model for eggshell thickness (EST) and eggshell strength (ESS). Plot A: regional and conditional plot for EST. Plot B: regional and conditional plot for ESS. (PDF 4277 kb) [file 12864_2015_1795_MOESM1_ESM.pdf]

Additional file 2:

Table S3 Summary of genetic analysis for eggshell thickness at different wks of age

Table S4 Summary of genetic analysis for eggshell strength at different wks of age

Table S5 Significant loci that located on 3’ or 5’ UTRs

Figure S1 Change curve of eggshell weight(ESW), eggshell percentage(ESP), eggshell thickness(EST) and eggshell strength(ESS) along with the age of laying hens. Plots A, B, C, D displayed the change curve of ESW, ESP, EST and ESS repectively.

Figure S2 Manhattan plot (left) and quantile-quantile plot (right) of the observed *P*-values for ESW, EST and ESS at age of first egg and at 32, 36, 40, 48, 52, 56, 60, 66, 72 wks of old. The Manhattan plot indicates $-\log_{10}$ (observed *P*-values) for genome-wide SNPs (y-axis) plotted against their respective positions on each chromosome (x-axis), and the horizontal green and black lines depict the genome-wide suggestive (1.69×10^{-5}) and significant (8.43×10^{-7}) threshold, respectively. For quantile-quantile plot, the x-axis shows the expected $-\log_{10}$-transformed *P*-values, and the y-axis represents the observed $-\log_{10}$-transformed *P*-values. The genomic inflation factors (λ) are shown on the top left in the QQ plot. Green points represent the genome-wide significant associations.

Figure S3 Regional plots and conditional analysis in multivariate model for eggshell thickness (EST) and eggshell strength (ESS). **Plot A**: regional and conditional plot for EST. **Plot B**: regional and conditional plot for ESS

Table S1. Summary of genetic analysis for eggshell thickness at different wks of age

Traits	FEST	EST32	EST36	EST40	EST44	EST48	EST52	EST56	EST60	EST66	EST72
**FEST**	0.26(0.04)	0.80(0.08)	0.86(0.09)	0.89(0.06)	0.73(0.10)	0.90(0.08)	0.85(0.08)	0.80(0.10)	0.75(0.10)	0.69(0.10)	0.63(0.11)
**EST32**	0.37	0.25(0.04)	0.93(0.06)	1.00(0.03)	1.00(0.04)	0.94(0.06)	1.00(0.06)	0.82(0.08)	0.76(0.09)	0.72(0.11)	0.80(0.09)
**EST36**	0.28	0.41	0.21(0.05)	0.94(0.05)	0.99(0.07)	0.95(0.08)	0.93(0.08)	0.80(0.09)	0.81(0.10)	0.80(0.11)	0.73(0.11)
**EST40**	0.37	0.56	0.45	0.31(0.04)	1.00(0.04)	1.00(0.04)	0.97(0.04)	0.89(0.06)	0.85(0.09)	0.79(0.09)	0.94(0.07)
**EST44**	0.30	0.48	0.37	0.52	0.24(0.05)	1.00(0.06)	1.00(0.05)	1.00(0.06)	0.86(0.08)	0.78(0.10)	1.00(0.07)
**EST48**	0.26	0.43	0.31	0.48	0.38	0.24(0.05)	0.94(0.07)	0.87(0.08)	0.85(0.07)	0.87(0.08)	0.89(0.08)
**EST52**	0.31	0.41	0.33	0.48	0.46	0.39	0.26(0.04)	1.00(0.06)	0.88(0.08)	0.84(0.09)	1.00(0.07)
**EST56**	0.23	0.38	0.30	0.44	0.40	0.39	0.44	0.24(0.03)	0.95(0.08)	0.88(0.09)	0.91(0.08)
**EST60**	0.28	0.37	0.33	0.47	0.42	0.40	0.45	0.47	0.25(0.04)	0.97(0.06)	0.60(0.07)
**EST66**	0.28	0.26	0.24	0.27	0.29	0.25	0.30	0.28	0.33	0.26(0.05)	0.94(0.06)
**EST72**	0.22	0.35	0.29	0.40	0.36	0.34	0.35	0.40	0.45	0.31	0.25(0.04)

Diagonal: heritability estimates. Lower triangle: phenotypic correlations. Upper triangle: genetic correlations. Standard errors of the estimates are in parentheses.

[a]FEST = eggshell thickness of first egg; EST32, EST36, EST40, EST44, EST48, EST52, EST56, EST60, EST66, EST72 = eggshell thickness of 32,36,40,44,48,52,56,60,66,72 wks of age.

Table S2. Summary of genetic analysis for eggshell strength at different wks of age

Traits	FESS	ESS32	ESS36	ESS40	ESS44	ESS48	ESS52	ESS56	ESS60	ESS66	ESS72
**FESS**	0.24(0.04)	0.71(0.10)	0.77(0.10)	0.7(0.10)	0.71(0.11)	0.71(0.11)	0.55(0.12)	0.67(0.10)	0.52(0.13)	0.69(0.10)	0.58(0.12)
**ESS32**	0.34	0.21(0.04)	1.00(0.07)	0.98(0.05)	0.98(0.05)	0.87(0.09)	0.84(0.09)	0.82(0.08)	0.82(0.10)	0.71(0.10)	0.64(0.11)
**ESS36**	0.30	0.47	0.20(0.04)	0.96(0.06)	1.00(0.07)	0.79(0.10)	0.85(0.09)	0.83(0.09)	0.87(0.09)	0.85(0.09)	0.88(0.08)
**ESS40**	0.29	0.53	0.49	0.31(0.04)	1.00(0.05)	0.96(0.05)	0.95(0.05)	0.92(0.05)	0.91(0.07)	0.93(0.06)	0.83(0.07)
**ESS44**	0.22	0.49	0.39	0.52	0.20(0.04)	1.00(0.07)	1.00(0.08)	1.00(0.07)	1.00(0.08)	0.92(0.09)	1.00(0.07)
**ESS48**	0.22	0.41	0.37	0.47	0.46	0.22(0.04)	0.92(0.07)	0.95(0.06)	0.92(0.08)	0.83(0.08)	0.88(0.07)
**ESS52**	0.24	0.43	0.35	0.51	0.48	0.50	0.27(0.05)	0.98(0.05)	0.99(0.07)	0.86(0.08)	0.92(0.07)
**ESS56**	0.21	0.35	0.33	0.45	0.41	0.48	0.50	0.27(0.04)	1.00(0.06)	0.96(0.06)	0.97(0.05)
**ESS60**	0.21	0.35	0.32	0.46	0.44	0.47	0.46	0.49	0.27(0.06)	1.00(0.07)	1.00(0.06)
**ESS66**	0.21	0.35	0.35	0.41	0.36	0.39	0.42	0.48	0.48	0.26(0.04)	1.00(0.05)
**ESS72**	0.22	0.40	0.35	0.46	0.42	0.43	0.44	0.54	0.54	0.54	0.27(0.05)

Diagonal: heritability estimates. Lower triangle: phenotypic correlations. Upper triangle: genetic correlations. Standard errors of the estimates are in parentheses.

[a]FESS = eggshell strength of first egg; ESS32, ESS36, ESS40, ESS44, ESS48, ESS52, ESS56, ESS60, ESS66, ESS72 = eggshell strength of 32,36,40,44,48,52,56,60,66,72 wks of age.

Table S3. Significant loci that located on 3’ or 5’ UTRs

Tag SNP	Chromosome	Positon	Location	Gene symbol
rs10725104	GGA1	59674042	3’UTR	DENND5B
rs14833862	GGA1	61107904	3’UTR	ADIPOR2
rs313549183	GGA1	61159783	3’UTR	LRTM2
rs314693803	GGA1	62316927	3’UTR	BPGM
rs15297718	GGA1	65450286	5’UTR	PYROXD1
rs313721110	GGA1	65469927	3’UTR	RECQL
rs313195183	GGA1	66990640	3’UTR	CMAS
rs317577390	GGA1	66991612		
rs318044141	GGA1	67332734	3’UTR	LYRM5
rs316447591	GGA1	67808349	3’UTR	ITPR2
rs316500020	GGA1	68144149	3’UTR	FGFR1OP2
rs318191682	GGA1	69350426	3’UTR	PARVG
rs314821358	GGA1	70396651	3’UTR	KIAA0930
rs314893458	GGA1	70533938	3’UTR	SMC1B
rs314893458	GGA1	70533938		

Figure S1

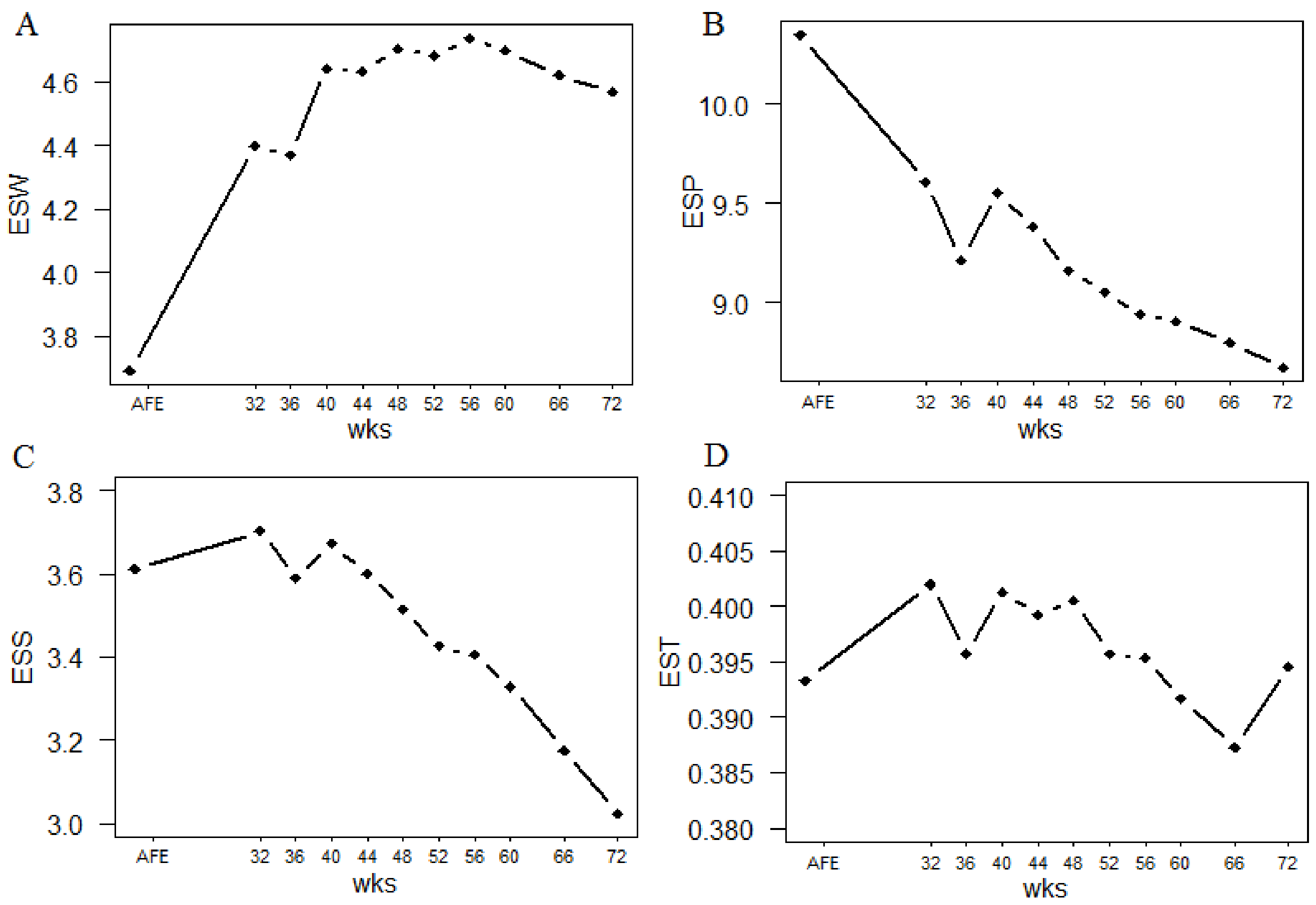

A
ESW
4.6
4.4
4.2
4.0
3.8
AFE
32
36
40
44
48
52
56
60
66
72
wks
B
ESP
10.0
9.5
9.0
C
ESS
3.8
3.6
3.4
3.2
3.0
D
EST
0.410
0.405
0.400
0.395
0.390
0.385
0.380

Figure S2

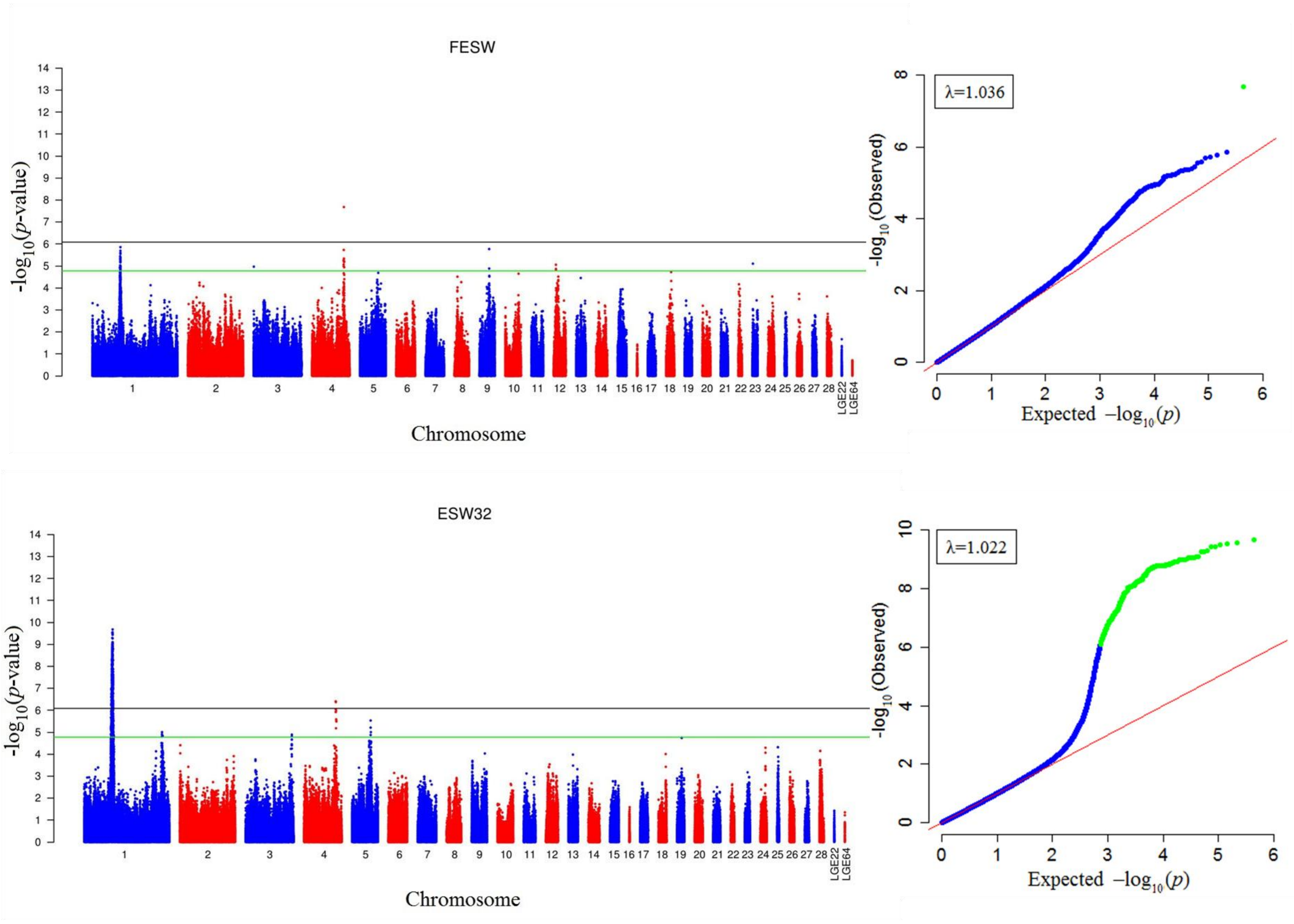

FESW
-log10(p-value)
Chromosome
λ=1.036
-log10(Observed)
Expected −log10(p)
ESW32
-log10(p-value)
Chromosome
λ=1.022
-log10(Observed)
Expected −log10(p)

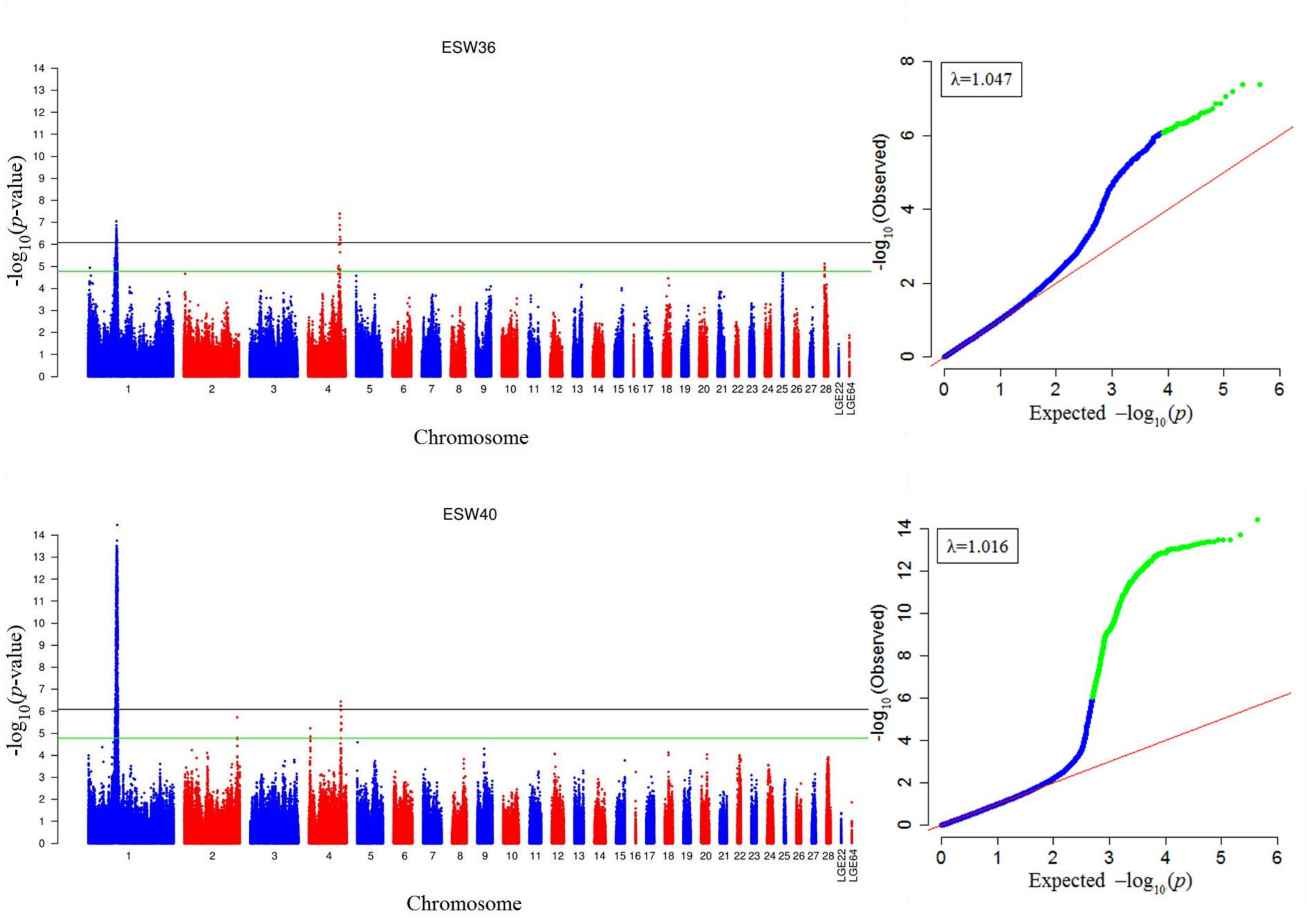
ESW36
-log10(p-value)
Chromosome
λ=1.047
-log10(Observed)
Expected −log10(p)
ESW40
-log10(p-value)
Chromosome
λ=1.016
-log10(Observed)
Expected −log10(p)
LGE22
LGE64

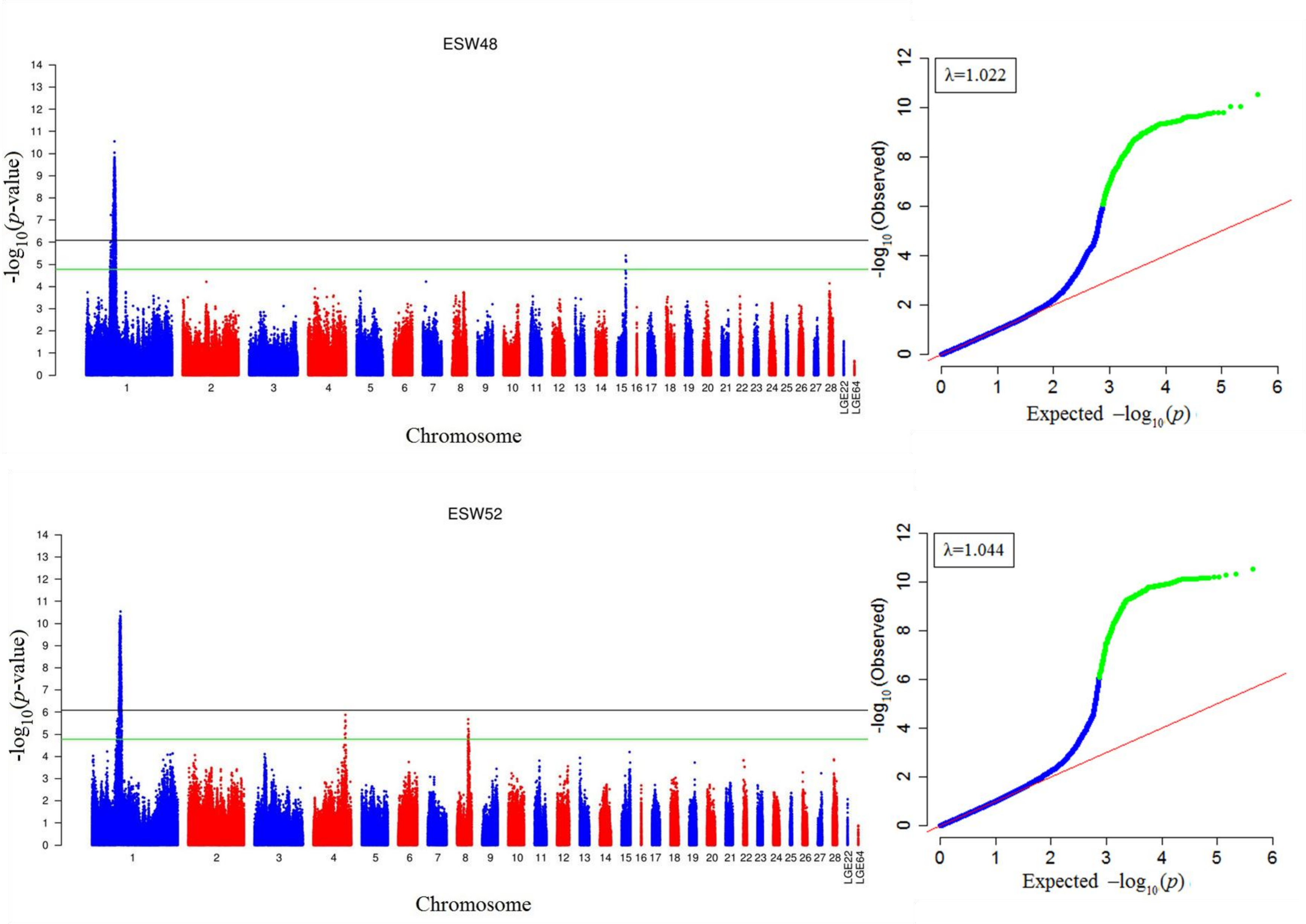

ESW48
-log10(p-value)
Chromosome
1 2 3 4 5 6 7 8 9 10 11 12 13 14 15 16 17 18 19 20 21 22 23 24 25 26 27 28 LGE22 LGE64
λ=1.022
-log10(Observed)
Expected −log10(p)
ESW52
-log10(p-value)
Chromosome
1 2 3 4 5 6 7 8 9 10 11 12 13 14 15 16 17 18 19 20 21 22 23 24 25 26 27 28 LGE22 LGE64
λ=1.044
-log10(Observed)
Expected −log10(p)

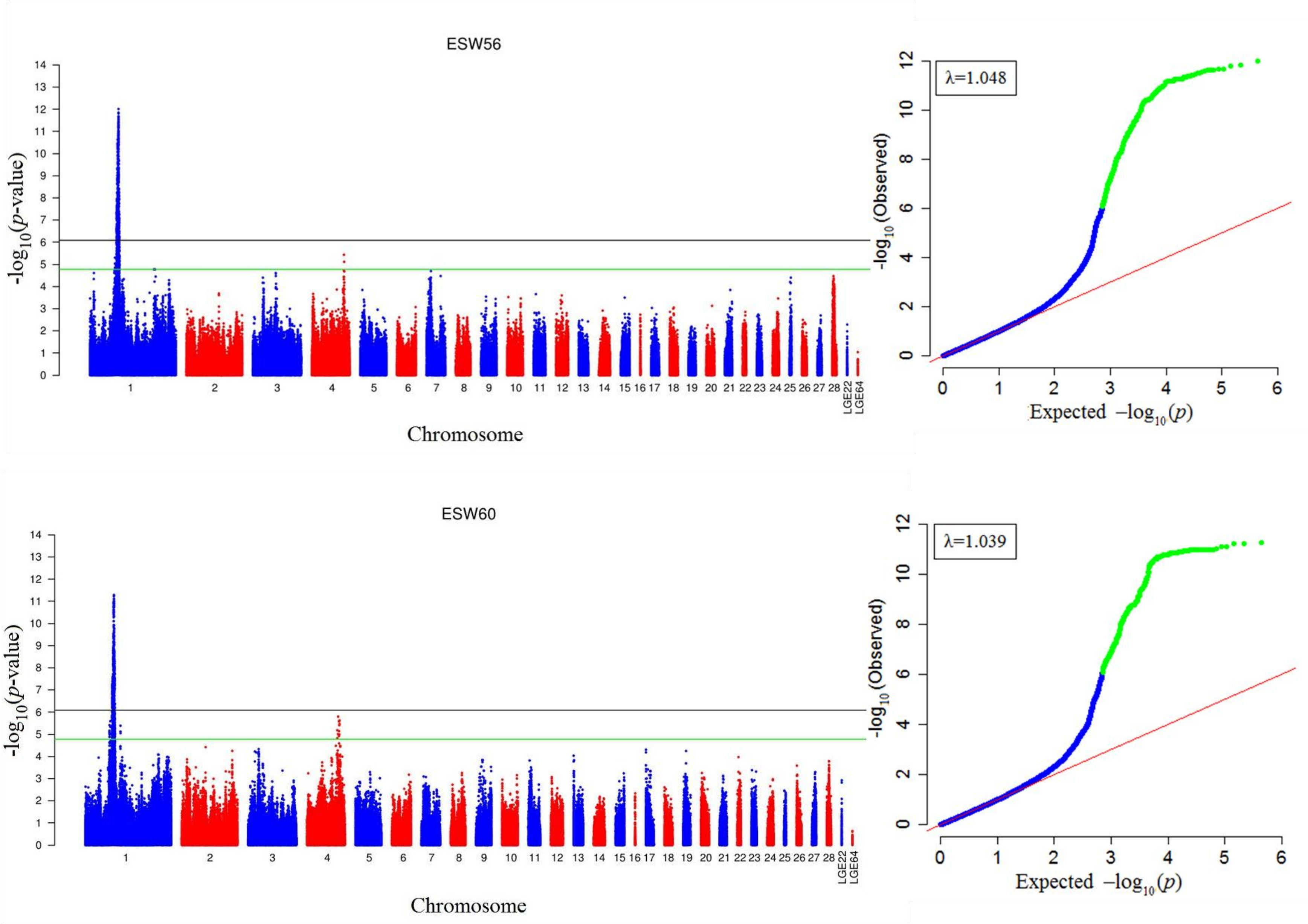
ESW56
-log10(p-value)
Chromosome
1 2 3 4 5 6 7 8 9 10 11 12 13 14 15 16 17 18 19 20 21 22 23 24 25 26 27 28 LGE22 LGE64
λ=1.048
-log10(Observed)
Expected −log10(p)
ESW60
-log10(p-value)
Chromosome
1 2 3 4 5 6 7 8 9 10 11 12 13 14 15 16 17 18 19 20 21 22 23 24 25 26 27 28 LGE22 LGE64
λ=1.039
-log10(Observed)
Expected −log10(p)

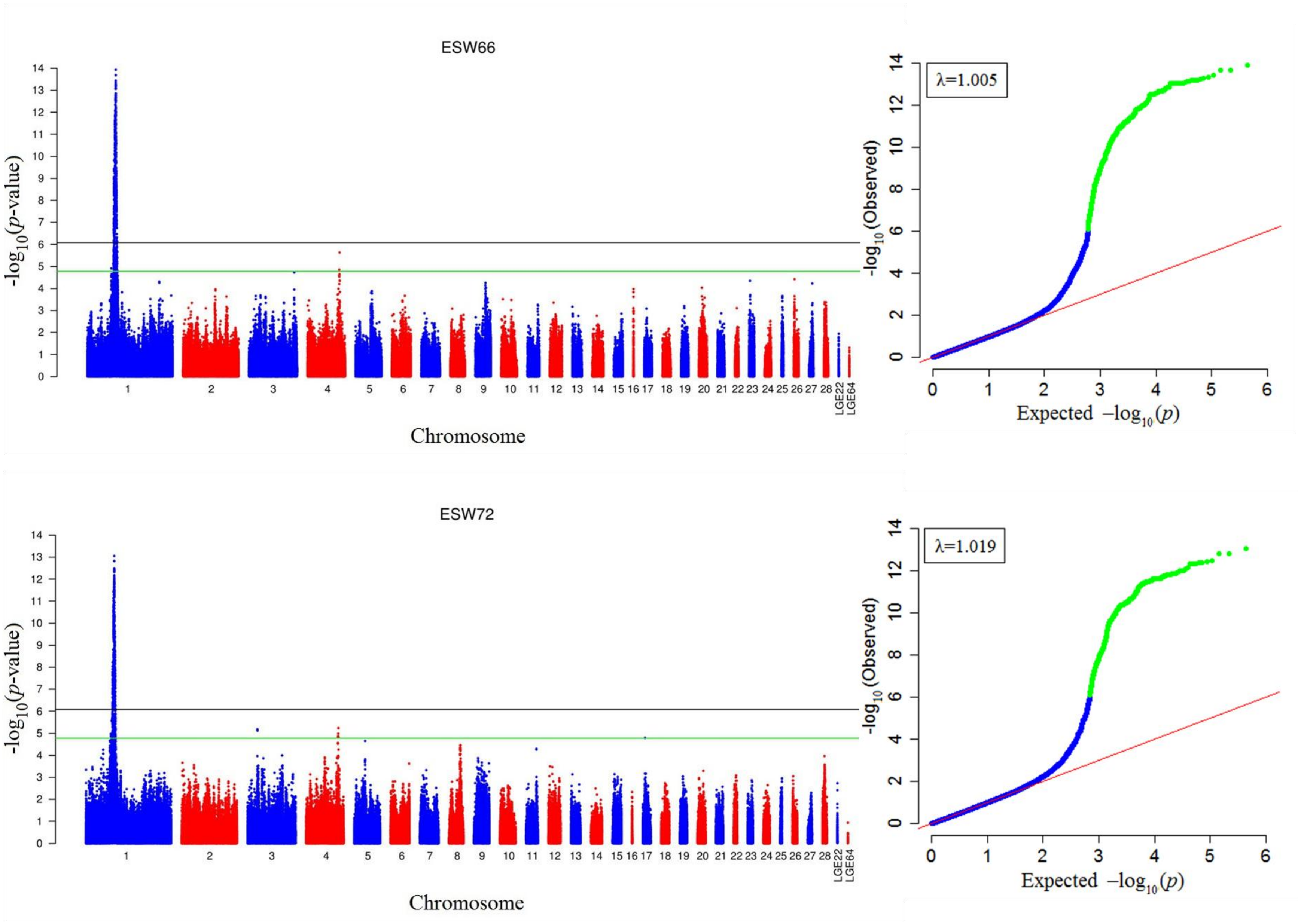
ESW66
-log10(p-value)
Chromosome
λ=1.005
-log10(Observed)
Expected −log10(p)
ESW72
-log10(p-value)
Chromosome
λ=1.019
-log10(Observed)
Expected −log10(p)

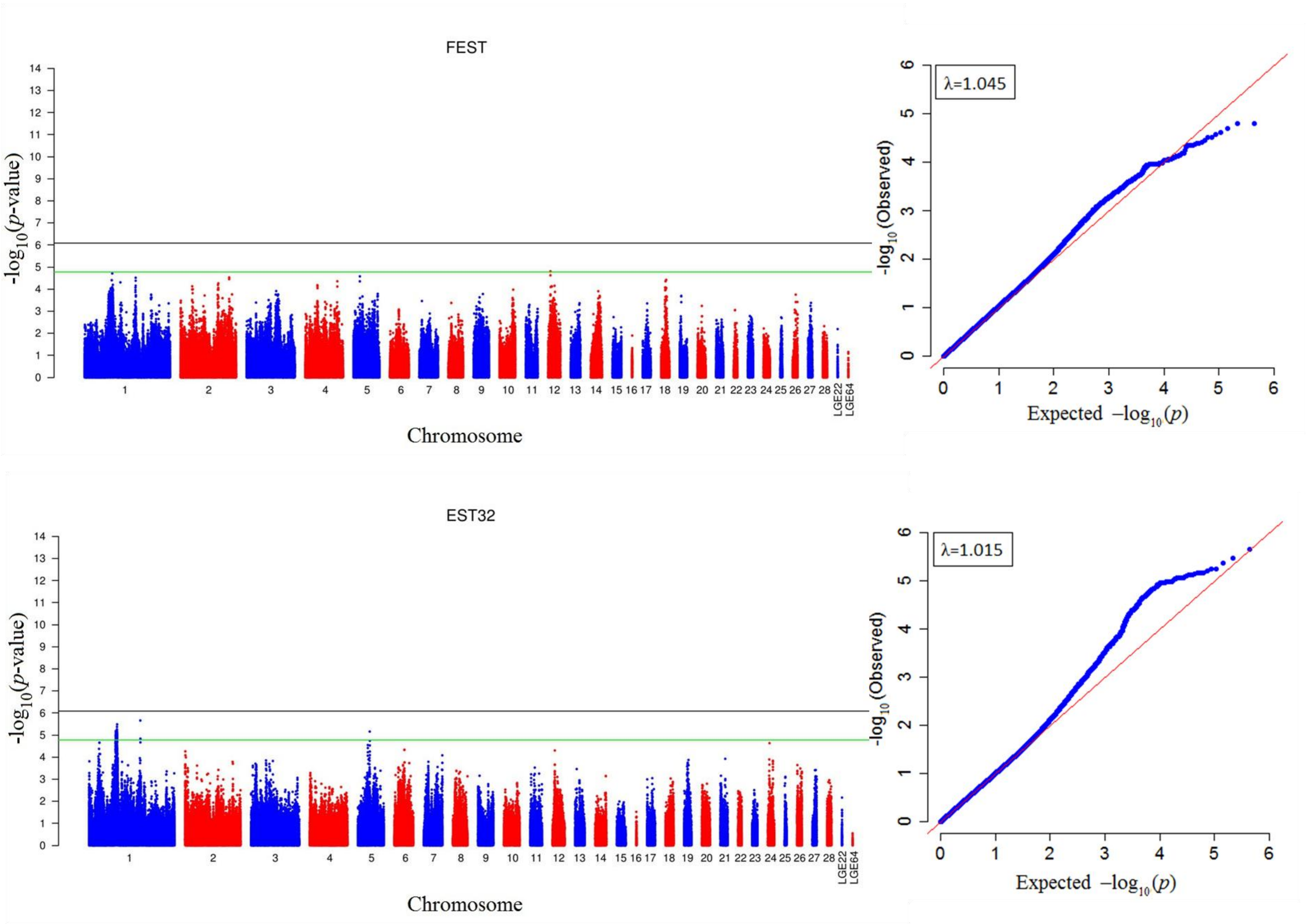
FEST
-log10(p-value)
Chromosome
LGE22
LGE64
λ=1.045
-log10(Observed)
Expected –log10(p)
EST32
-log10(p-value)
Chromosome
LGE22
LGE64
λ=1.015
-log10(Observed)
Expected –log10(p)

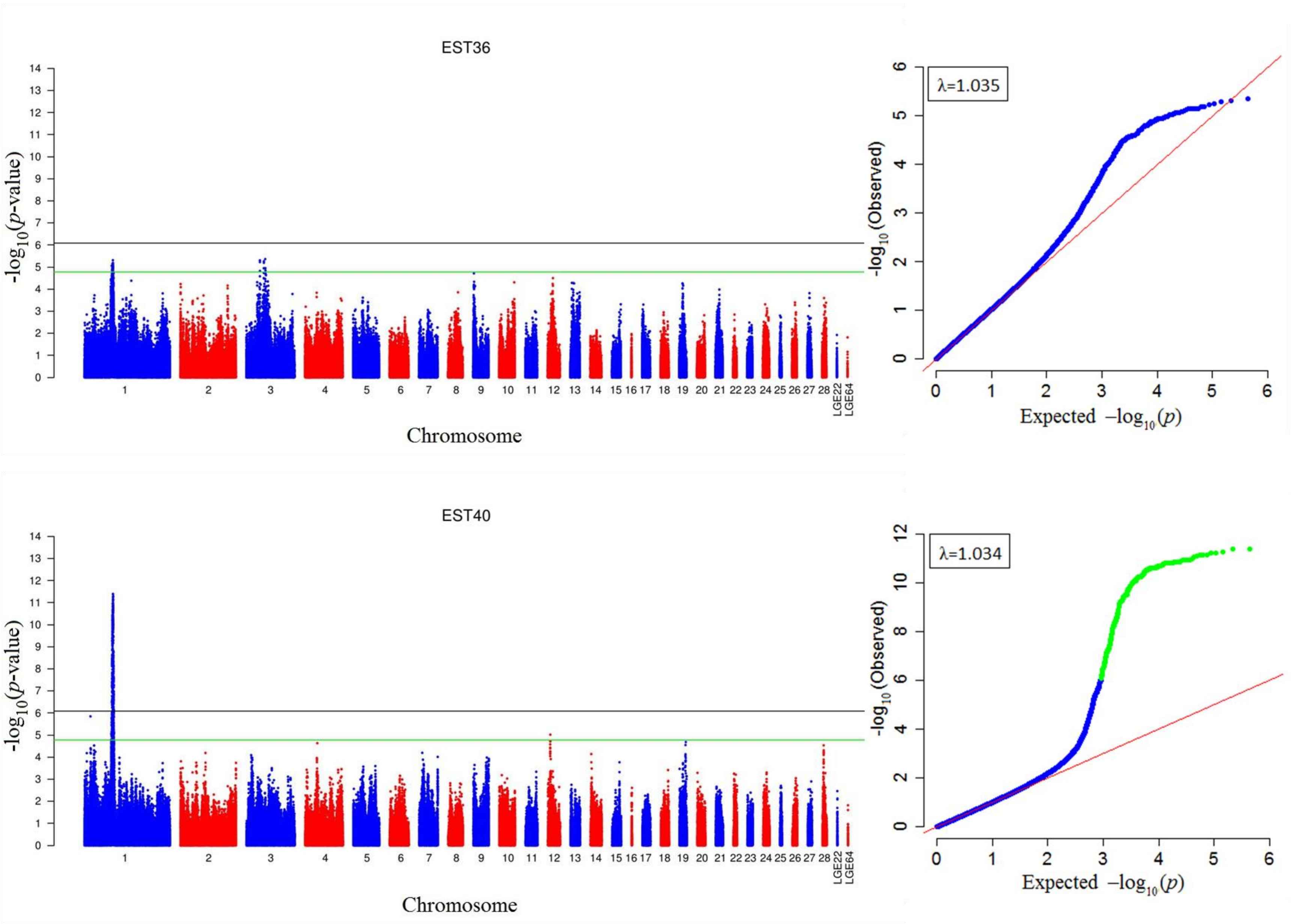
EST36
-log10(p-value)
Chromosome
λ=1.035
-log10(Observed)
Expected –log10(p)
EST40
-log10(p-value)
Chromosome
λ=1.034
-log10(Observed)
Expected –log10(p)

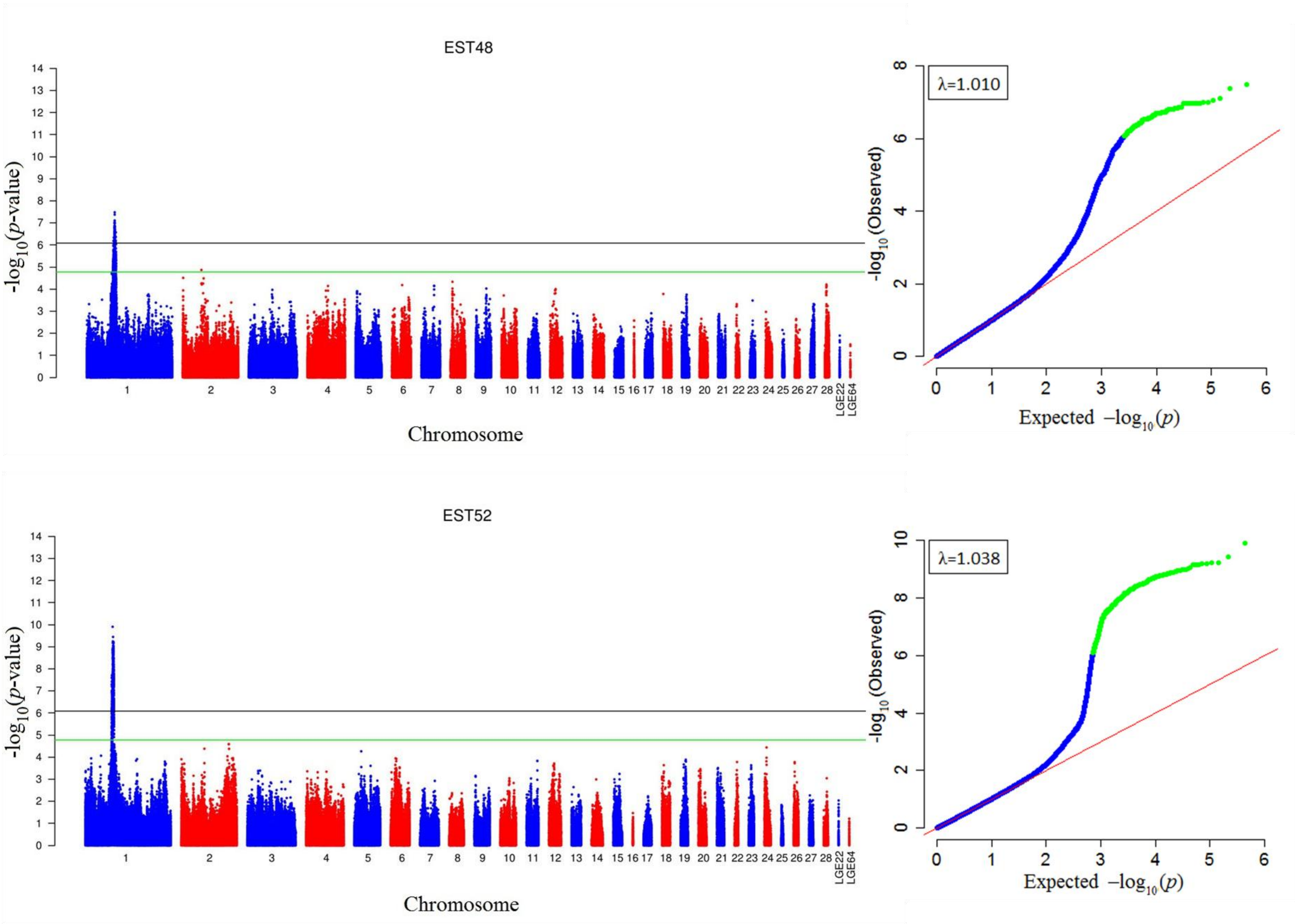
EST48
-log10(p-value)
Chromosome
λ=1.010
-log10(Observed)
Expected −log10(p)
EST52
-log10(p-value)
Chromosome
λ=1.038
-log10(Observed)
Expected −log10(p)
LGE22
LGE64

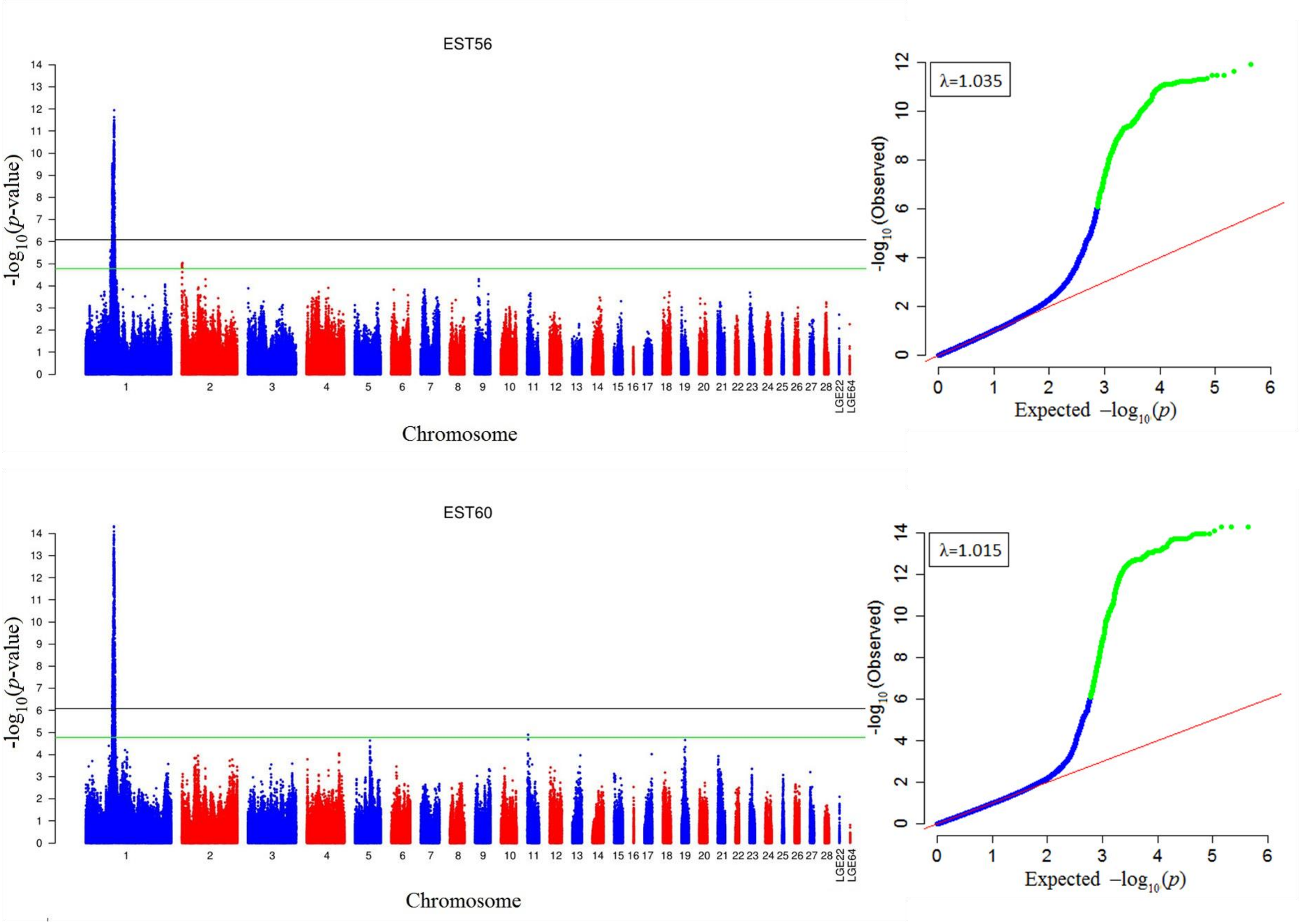
EST56
-log10(p-value)
Chromosome
λ=1.035
-log10 (Observed)
Expected −log10(p)
EST60
-log10(p-value)
Chromosome
λ=1.015
-log10 (Observed)
Expected −log10(p)

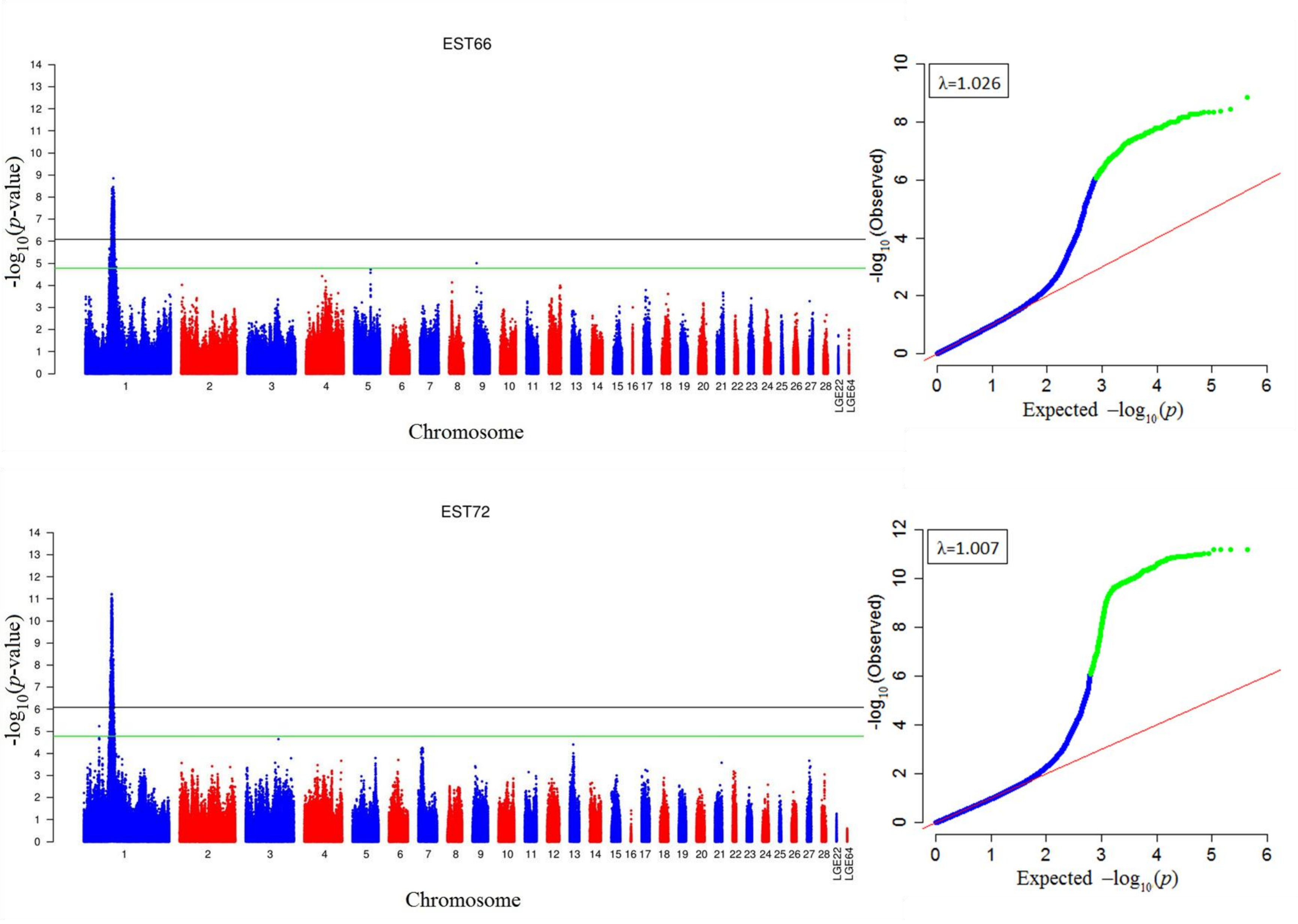
EST66
$-\log_{10}(p\text{-value})$
Chromosome
LGE22
LGE64
λ=1.026
$-\log_{10}$(Observed)
Expected $-\log_{10}(p)$
EST72
$-\log_{10}(p\text{-value})$
Chromosome
LGE22
LGE64
λ=1.007
$-\log_{10}$(Observed)
Expected $-\log_{10}(p)$

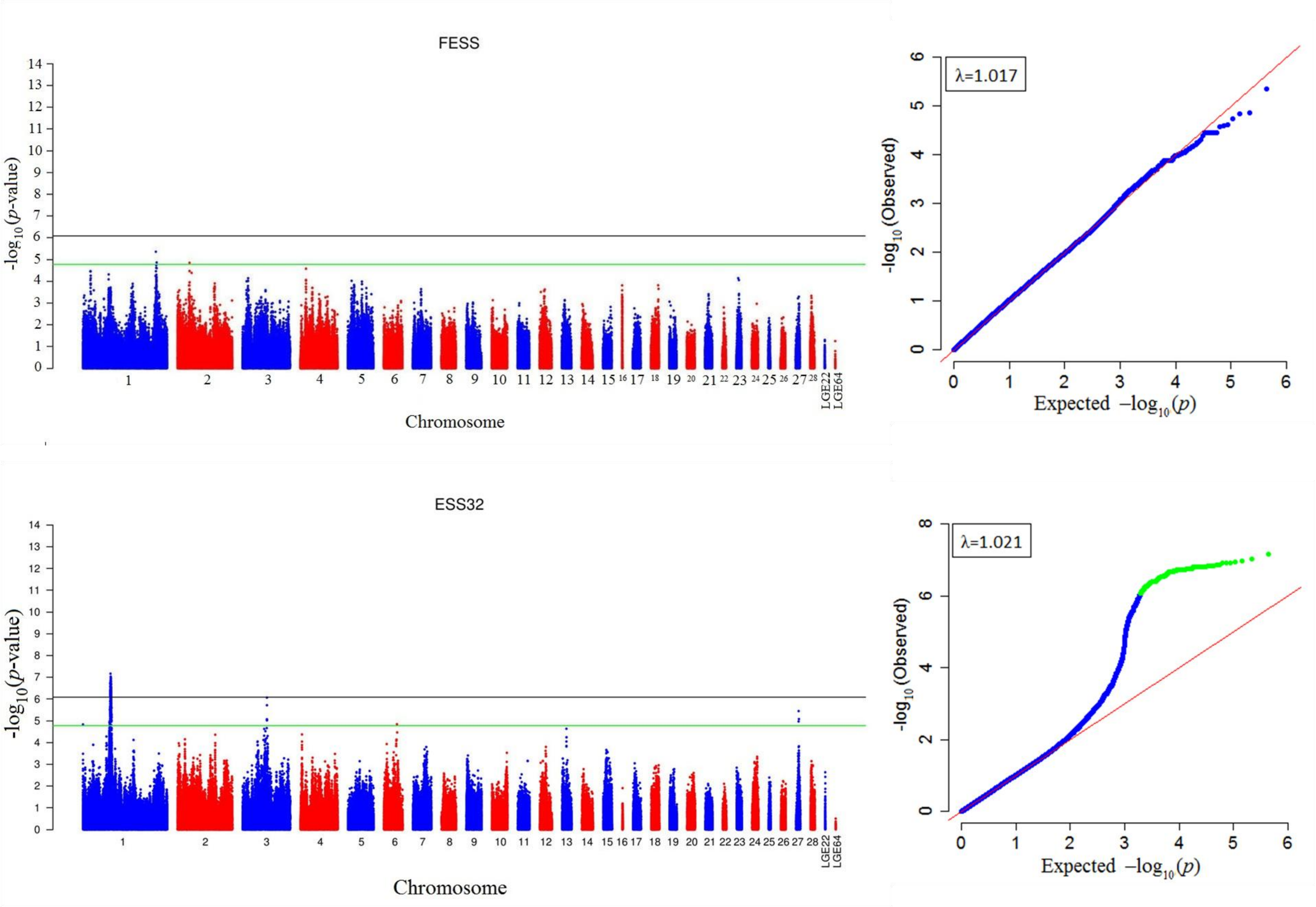
FESS
-log10(p-value)
Chromosome
LGE22
LGE64
λ=1.017
-log10(Observed)
Expected −log10(p)
ESS32
-log10(p-value)
Chromosome
LGE22
LGE64
λ=1.021
-log10(Observed)
Expected −log10(p)

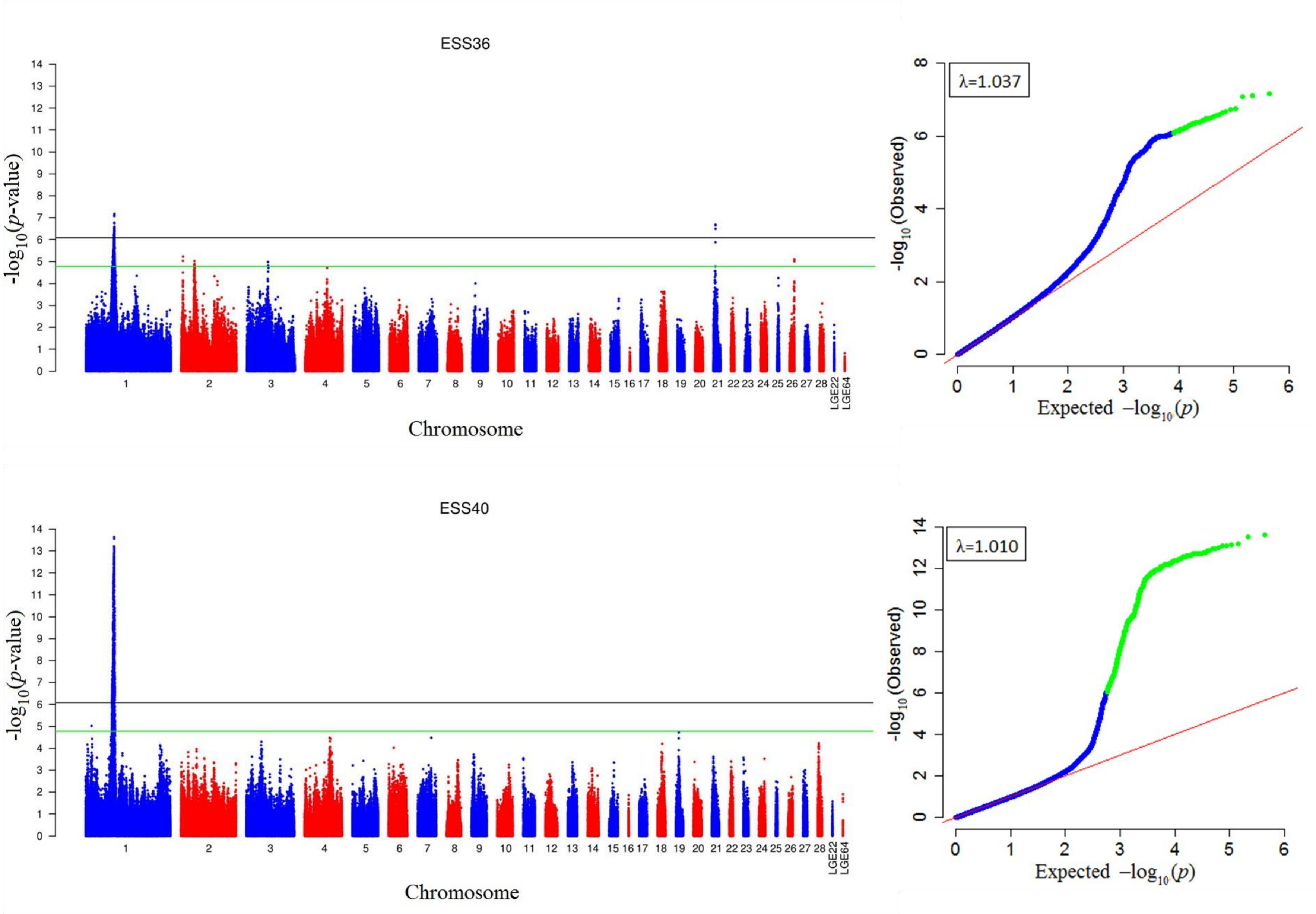
ESS36
-log10(p-value)
Chromosome
λ=1.037
-log10(Observed)
Expected −log10(p)
ESS40
-log10(p-value)
Chromosome
λ=1.010
-log10(Observed)
Expected −log10(p)

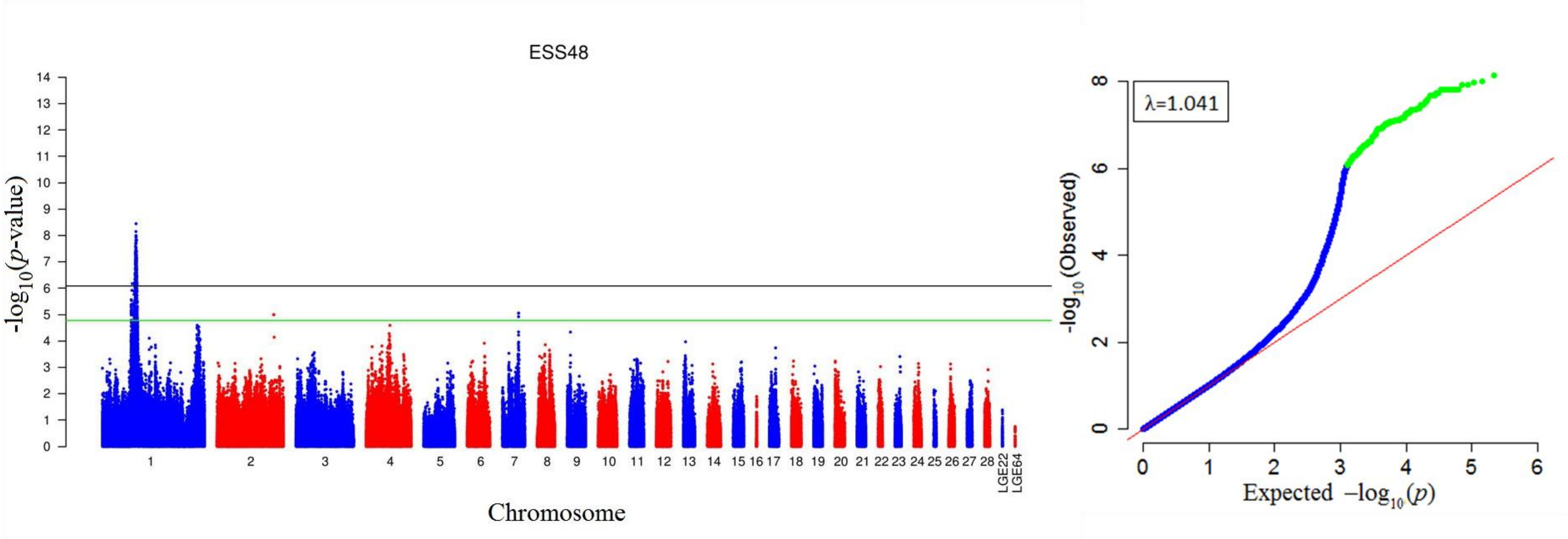
ESS48
-log10(p-value)
Chromosome
1 2 3 4 5 6 7 8 9 10 11 12 13 14 15 16 17 18 19 20 21 22 23 24 25 26 27 28 LGE22 LGE64
λ=1.041
-log10(Observed)
Expected −log10(p)

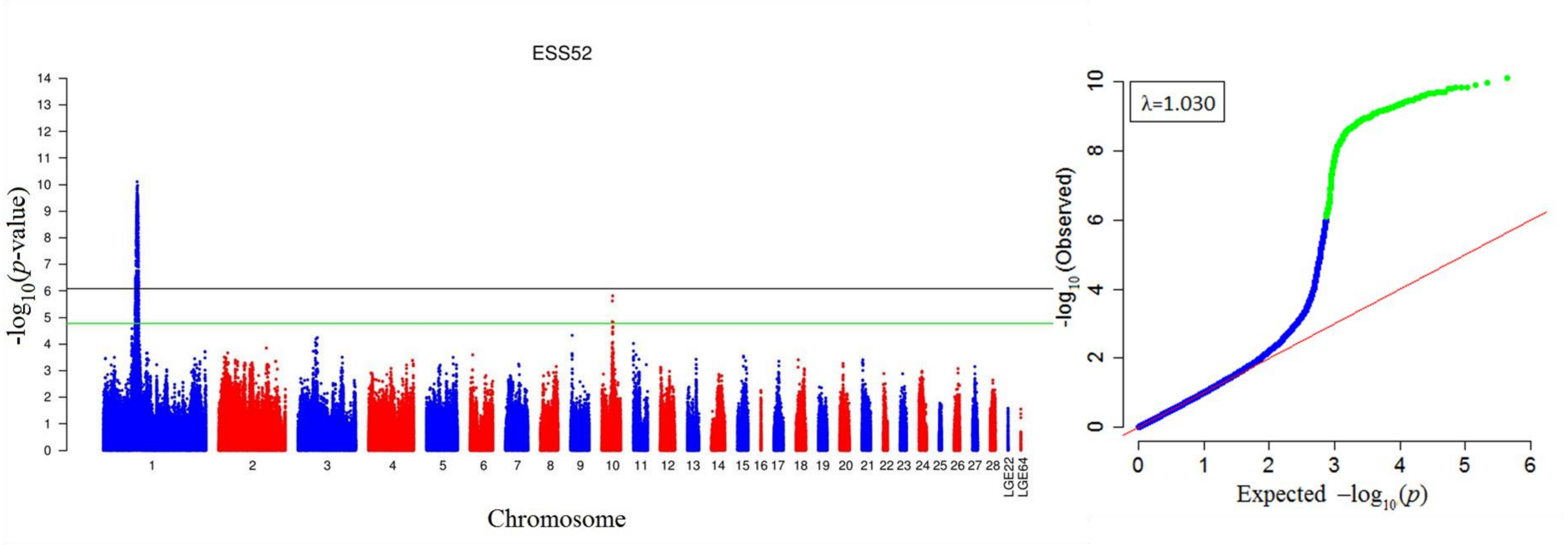
ESS52
-log10(p-value)
Chromosome
1 2 3 4 5 6 7 8 9 10 11 12 13 14 15 16 17 18 19 20 21 22 23 24 25 26 27 28 LGE22 LGE64
λ=1.030
-log10(Observed)
Expected −log10(p)

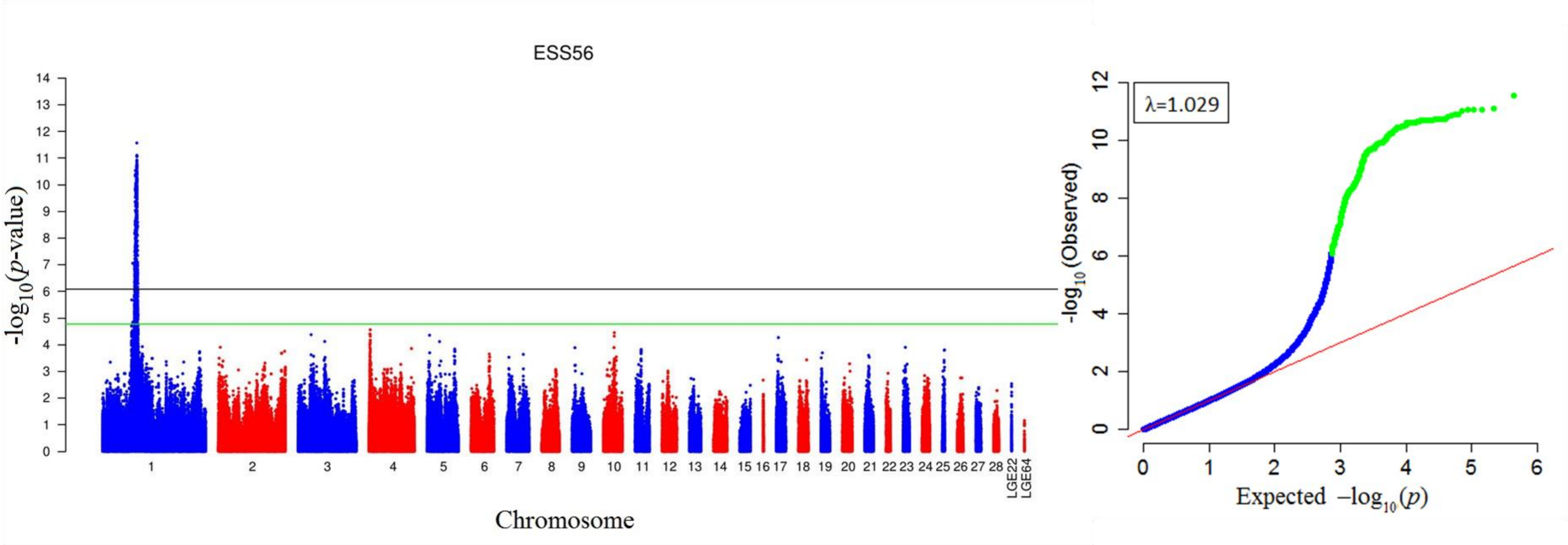
ESS56
-log10(p-value)
Chromosome
λ=1.029
-log10(Observed)
Expected −log10(p)

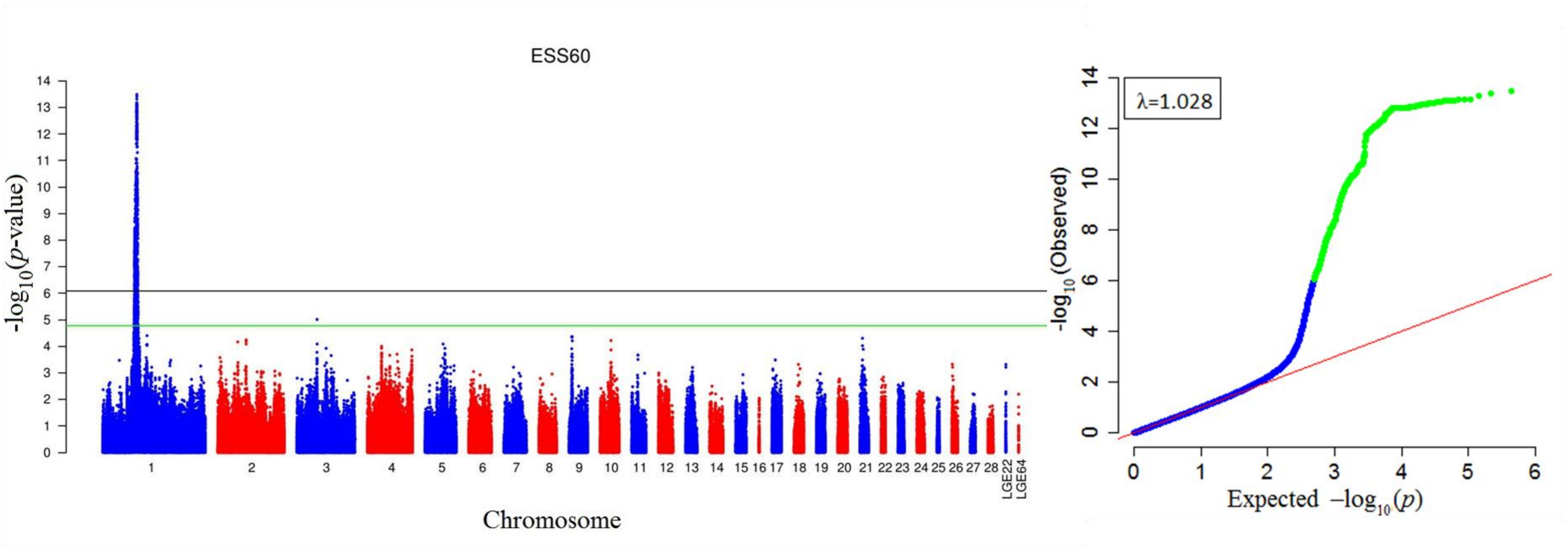
ESS60
-log10(p-value)
Chromosome
λ=1.028
-log10(Observed)
Expected −log10(p)

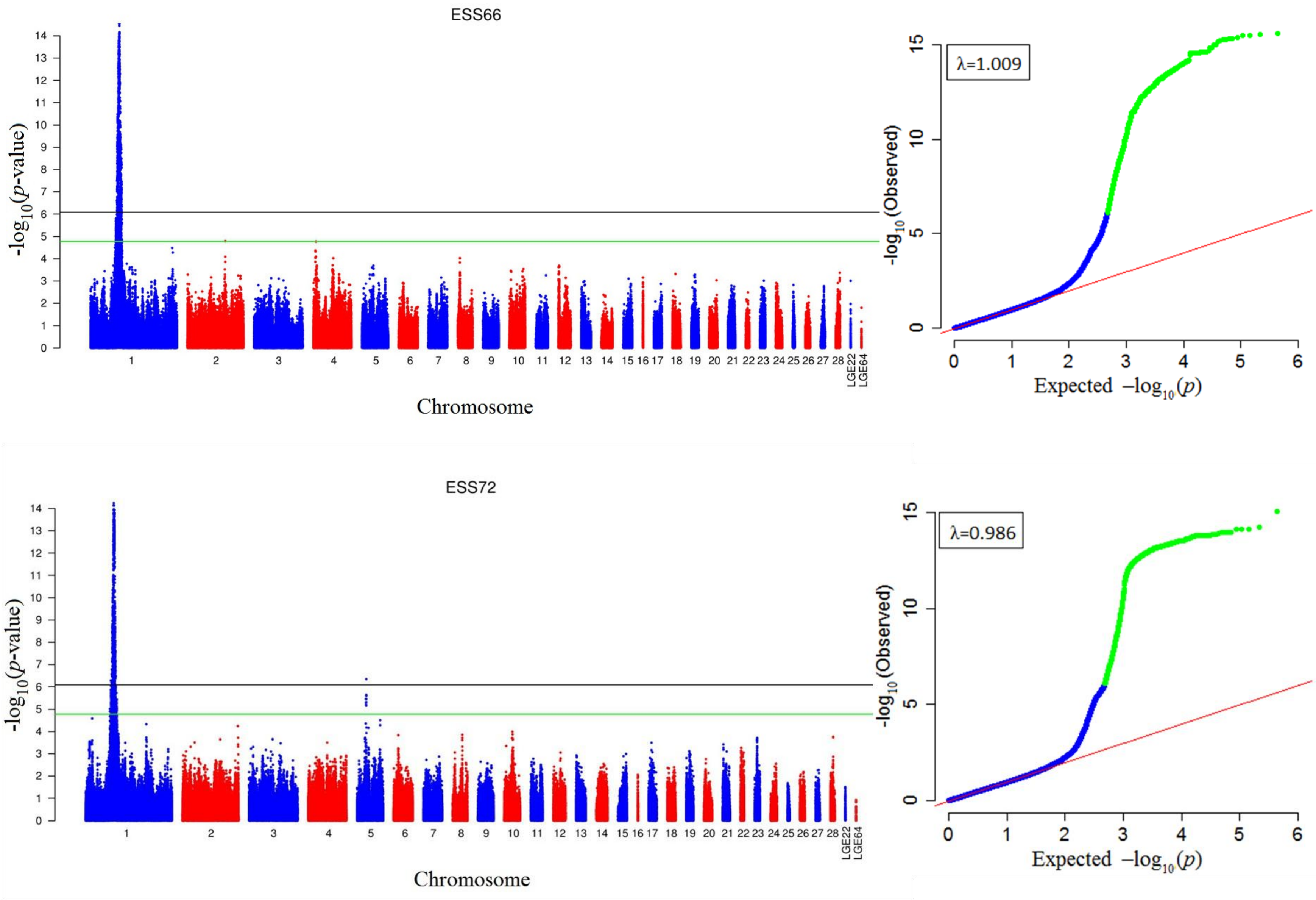
ESS66
-log10(p-value)
Chromosome
λ=1.009
-log10(Observed)
Expected −log10(p)
ESS72
-log10(p-value)
Chromosome
λ=0.986
-log10(Observed)
Expected −log10(p)

Figure S3

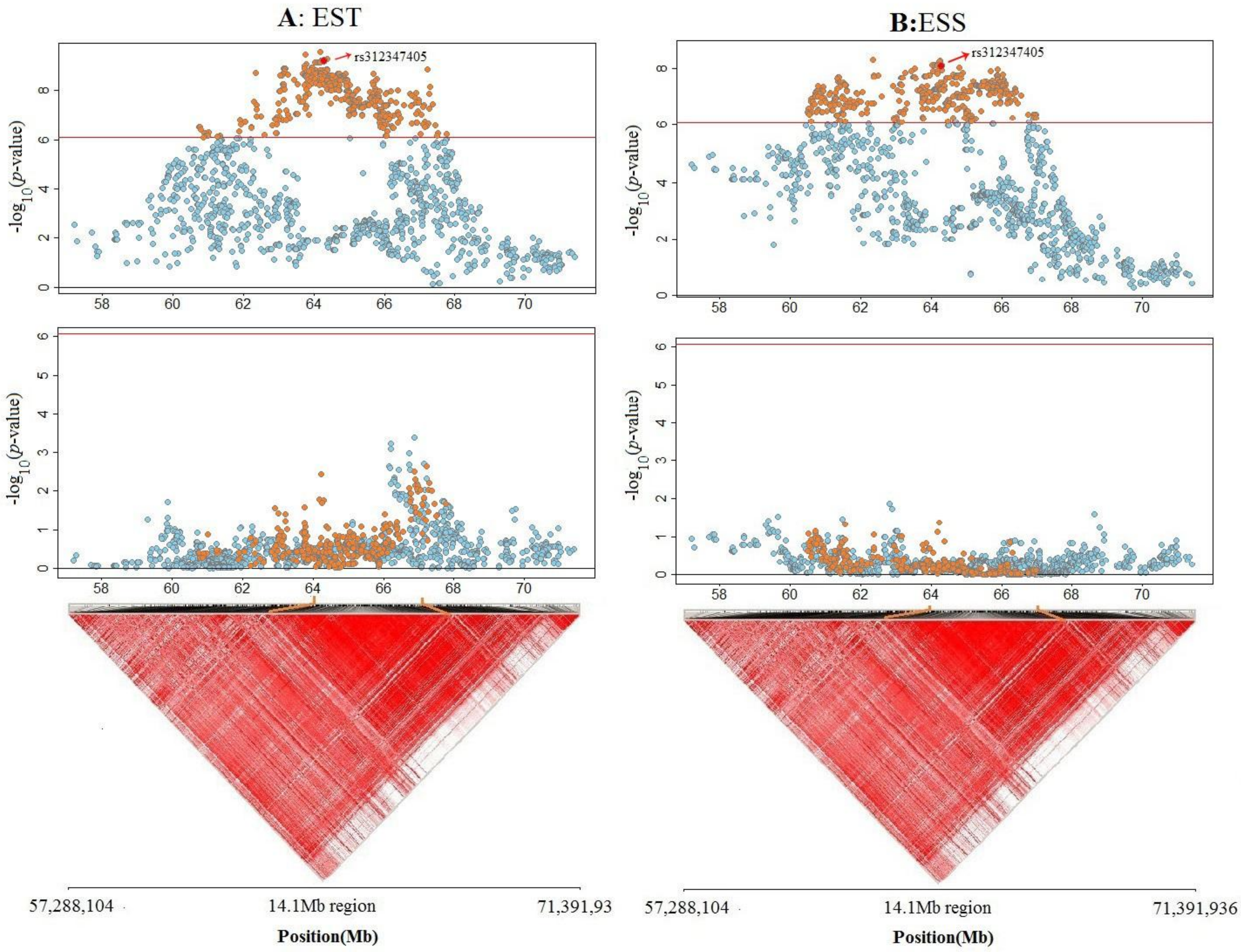
A: EST
B:ESS
rs312347405
rs312347405
-log10(p-value)
57,288,104
14.1Mb region
71,391,93
57,288,104
14.1Mb region
71,391,936
Position(Mb)
Position(Mb)
